# EHR foundation models improve robustness in the presence of temporal distribution shift

**DOI:** 10.1038/s41598-023-30820-8

**Published:** 2023-03-07

**Authors:** Lin Lawrence Guo, Ethan Steinberg, Scott Lanyon Fleming, Jose Posada, Joshua Lemmon, Stephen R. Pfohl, Nigam Shah, Jason Fries, Lillian Sung

**Affiliations:** 1 Program in Child Health Evaluative Sciences, The Hospital for Sick Children, Toronto, ON Canada; 2 Stanford Center for Biomedical Informatics Research, Stanford University, Palo Alto, CA USA; 3 Universidad del Norte, Barranquilla, Colombia; 4 Division of Haematology/Oncology, The Hospital for Sick Children, 555 University Avenue, Toronto, ON M5G1X8 Canada

**Keywords:** Health care, Machine learning

## Abstract

Temporal distribution shift negatively impacts the performance of clinical prediction models over time. Pretraining foundation models using self-supervised learning on electronic health records (EHR) may be effective in acquiring informative global patterns that can improve the robustness of task-specific models. The objective was to evaluate the utility of EHR foundation models in improving the in-distribution (ID) and out-of-distribution (OOD) performance of clinical prediction models. Transformer- and gated recurrent unit-based foundation models were pretrained on EHR of up to 1.8 M patients (382 M coded events) collected within pre-determined year groups (e.g., 2009–2012) and were subsequently used to construct patient representations for patients admitted to inpatient units. These representations were used to train logistic regression models to predict hospital mortality, long length of stay, 30-day readmission, and ICU admission. We compared our EHR foundation models with baseline logistic regression models learned on count-based representations (count-LR) in ID and OOD year groups. Performance was measured using area-under-the-receiver-operating-characteristic curve (AUROC), area-under-the-precision-recall curve, and absolute calibration error. Both transformer and recurrent-based foundation models generally showed better ID and OOD discrimination relative to count-LR and often exhibited less decay in tasks where there is observable degradation of discrimination performance (average AUROC decay of 3% for transformer-based foundation model vs. 7% for count-LR after 5–9 years). In addition, the performance and robustness of transformer-based foundation models continued to improve as pretraining set size increased. These results suggest that pretraining EHR foundation models at scale is a useful approach for developing clinical prediction models that perform well in the presence of temporal distribution shift.

## Introduction

The large increase in the adoption of electronic health records (EHR) has enabled the use of machine learning to develop highly performant clinical prediction models that have the potential to improve the care of patients^1^. However, the non-stationary healthcare environment can bring about changes in the data distribution between model development and deployment^2^, which can degrade the model’s performance over time^3^ and consequently its clinical utility^4^. In this study, we explored temporal distribution shift alongside the suitability of *foundation models*^5^—deep neural networks trained on large-scale unlabeled data using self-supervised learning—and whether they can be adapted via transfer learning to improve the robustness of clinical prediction models in the presence of temporal distribution shift.

The cause of temporal distribution shift in clinical medicine is often subtle^6^ and the extent of its impact on model performance is heterogeneous across tasks^3,7–9^. Nonetheless, the consequence of the impact on patient care and physician’s trust can be severe. An example is the widely implemented Epic sepsis model developed on data collected between 2013 and 2015 that performed below expectation when evaluated at Michigan Medicine on data collected between 2018 and 2019 and resulted in a large number of spurious alerts^4^.

Recent approaches that mitigate the impact of temporal distribution shift on model performance in clinical medicine largely rely on model monitoring and updating policies that do not leverage the entire patient population available^10^. In addition, proactive approaches using domain generalization and adaptation have shown little to no success^3^.

While recent work on medical foundation models has focused on improving sample complexity when fine-tuning, little-to-no work has measured a pretrained, medical foundation model’s impact on temporal robustness in clinical prediction tasks. Findings from domains outside of clinical medicine suggest significant performance^11^ and robustness^12,13^ benefits to pretraining foundation models, and these benefits tend to increase with scale^14,15^. Another major benefit of foundation models is their ability to generalize to tasks not seen during training^16^. In this study, we adopt EHR foundation models—deep neural networks pretrained on EHR-derived patient timelines using self-supervised learning. Patient timelines consist of structured medical codes ordered by time, where each code (e.g., M32.9 for “lupus erythematosus”) functions as a word drawn from a finite vocabulary defined by medical ontologies such as ICD10. This formulation enables using autoregressive sequence modeling, a self-supervised learning objective used in natural language processing, to train an EHR foundation model by predicting the next day’s codes. The resulting pretrained model is then used to generate feature representations for downstream tasks. The foundation modeling approach in this study is referred to as *clinical language model based representations* (CLMBR)^17^. Transfer of the structure learned by CLMBR from the entire patient population to downstream clinical prediction models have demonstrated performance benefits compared to standard baselines including count-based models, especially when the number of patient records was small^17^. CLMBR’s architecture aligns with other EHR foundation models, such as Med-BERT^18^ and BEHRT^19^, but uses an autoregressive instead of masked language modeling objective for pretraining to match the next-day prediction task. We refer to CLMBR as an EHR foundation model because of its potential to shift practice in the development of machine learning models for clinical medicine. We focus specifically on the implications for temporal robustness when adapting task-specific models from a shared, self-supervised model trained on a patient population. However, we recognize that scale (both in parameter count and training data size) is a key aspect of modern foundation models and that structured EHR models are currently much smaller than their counterparts in language and vision. For example, GPT-3^16^ has 175 billion parameters compared to 42 million for the largest CLMBR model used in this study. Thus our work is more akin to early NLP foundation models such as BERT^11^ or ELMO^20^.

We evaluated the utility of CLMBR in mitigating the impact of temporal distribution shift on model performance. We hypothesized that the global patterns embedded in CLMBR can be adapted into models that perform better than count-based representations in out-of-distribution (OOD) years. For adaptation, we froze CLMBR weights and trained a classification head using logistic regression (CLMBR-LR) for each downstream clinical prediction task. The primary objective was to compare the in-distribution (ID) and OOD performance of CLMBR-LR to models trained on count-based representations. In addition, we examined the contribution of CLMBR pretraining and characterized the performance and robustness of different architecture choices for CLMBR (gated recurrent units and transformers) as well as how the performance of each architecture scales with increasing quantity of pretraining data.

## Methods

### Data source

We used data from the STAnford medicine Research data Repository (STARR)^21^. Data in STARR are routinely collected in the EHR of Stanford Medical Center, comprised of Stanford Health Care (primarily adult-directed care) and Lucile Packard Children’s Hospital (primarily pediatric-directed care). These data are mapped to the Observational Medical Outcomes Partnership Common Data Model (OMOP-CDM), which facilitates multi-center observational research^22,23^. This study used de-identified data in which dates within a patient timeline were jittered by up to 30 days, but their temporal relations remained intact. Because of de-identification, there was no requirement for Institutional Review Board approval and informed consent by Stanford Medical Center. Instead, access to data is restricted and subject to the data use agreement^21^. The use of this data and methods were carried out in accordance with relevant guidelines and regulations.

### Cohorts

We defined two types of cohorts: pretraining and task-specific. Pretraining cohorts contained patients on which CLMBR was pretrained using the self-supervised, autoregressive objective. Several pretraining cohorts were defined according to the experimental setups and differ in the number of patients included—from 36 K (42 M coded events) to 1.8 M (382 M coded events)—and the year range in which patient encounter days were considered for pretraining (see Fig. 2 and flow diagrams of patient cohort allocation in Supplementary Methods online for details). Note that while it is possible for a patient to have encounters beyond the year range specified for the pretraining cohort, these were excluded for pretraining. In addition, to be included in a pretraining cohort the patient must have had at least 3 patient days (of any encounter type) with clinical events during the time window defined by the year range.

The task-specific cohort contained patients on which classification heads for clinical prediction tasks were trained and evaluated. This cohort included adult patients over the age of 18 with admissions to the inpatient unit between EHR inception (2009) and August 22, 2021. Admissions to the inpatient unit were either direct or transfers from the emergency department. Encounters with clinic visits alone and encounters in which patient death or discharges occurred on the same day of admission were excluded. For patients with multiple admissions, one was randomly selected as the index admission for all tasks. Note that patients in the task-specific cohort may overlap with the pretraining cohorts, however patients in the validation and test sets of the task-specific cohort were excluded from all pretraining cohorts in order to prevent data leakage.

### Outcomes

We defined four clinical outcomes that are relevant for inpatient admissions for each patient in the task-specific cohort. *Hospital mortality* was defined as a patient death occurring during the index admission. Long length of stay (LOS) (*long LOS*) was defined as an index admission of seven or more days. Readmission in 30 days (*30-day readmission*) was defined as a readmission to an inpatient unit within 30 days after discharge. Intensive care unit (*ICU)* admission was defined as a patient transfer to the intensive care unit during the index admission. Each outcome was considered as a binary classification task where the prediction time was set as 11:59PM on the first day of the index admission for the hospital mortality, long LOS and ICU admission tasks, and 11:59PM on the day of discharge for the 30-day readmission prediction task. For the 30-day readmission task, we removed patients who were re-admitted on the day of discharge, and for the ICU admission task, we removed patients transferred on the first day of the index admission since these events would have occurred before prediction time.

### Patient representations

**Figure 1:**
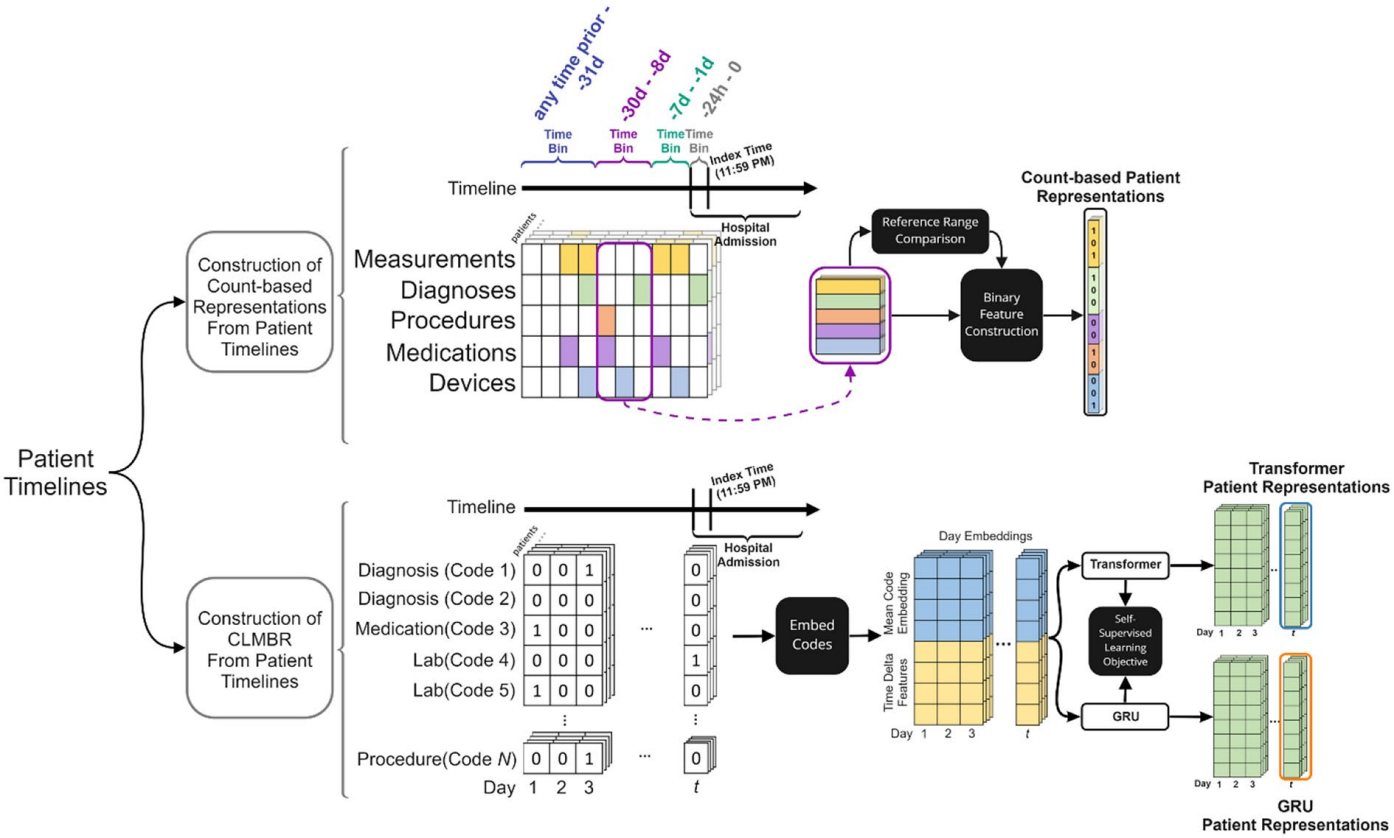
An overview of the two approaches of constructing patient representations used in this study. The purple box in the construction of count-based representations represents the reference range comparison and binary feature construction procedures for a specific time-bin. The construction of CLMBR illustrates the self-supervised pretraining stage, hence the inclusion of the self-supervised learning objective. The adaptation of CLMBR to specific tasks (e.g., for predicting hospital mortality) does not include the self-supervised learning objective. In addition, during adaptation CLMBR weights were frozen, and a separate classification head is learned on the same patient representations for each clinical prediction task. *CLMBR* Clinical language model-based representations.

EHR data corresponding to a particular patient can be treated as a sequence of days that is ordered by time, *d*_*1*_* … d*_*N*_, where each day consists of a set of events represented by medical codes such as diagnoses, lab tests, procedures, and medication administrations or prescriptions as examples. In this study, we considered two approaches to construct patient representations over the patient timelines as illustrated in Fig. 1: count-based representations and CLMBR.

#### Count based representations

The count-based representations were constructed for patients in the task-specific cohort using an open-source count-based featurizer^24^ which follows standard practices for patient count-based featurization^1,25^. This approach constructed patient representations as binary features based on counts of both unique OMOP CDM concepts and derived elements recorded prior to the time of prediction. The feature set consisted of demographic and clinical features. Demographic features included sex at birth, race, ethnicity, and age at admission discretized into 5-year intervals. Clinical features were constructed as the concatenation of the results of a time-dependent extraction procedure applied independently to data elements recorded in time bins defined relative to the time of prediction. The time bins were as follows: 24 h prior, 1–7 days prior, 8–30 days prior, and 31 days-any time prior. The time-dependent extraction procedure identified all unique OMOP CDM concepts from the following OMOP CDM tables: condition occurrence (diagnosis codes), drug exposure (administration or prescription of medications), procedure occurrence, measurement (includes laboratory tests), device exposure (exposure to implantable objects, medical equipment, supplies, and instruments) and observation (non-standardized tests or clinical observations). Continuous measurement results were represented as binary indicators for abnormal results for each measurement on the basis of whether the result was above or below the reference range in the EHR.

#### Clinical language model-based representations—CLMBR

The core idea behind CLMBR is that if a sequence model is able to predict sets of medical codes over a patient timeline, then that model may have discovered informative global patterns that can be re-used in various other clinical prediction tasks. Note that the term “language model” in CLMBR merely reflects the similarity in the computations involved between sequence modeling of medical codes and language modeling, and therefore does not indicate natural language processing of any kind.

First, we mapped clinical codes for labs, medications, diagnoses, and procedures to a finite vocabulary of discrete symbols. This vocabulary was then mapped into a clinical ontology to reduce sparsity and used to construct patient sequences for the CLMBR encoder. The medical codes were obtained from the same OMOP CDM tables as used for count-based representations except for the observation table. The Unified Medical Language System (UMLS)^26^ was used to extend each medical code to the set of parents in its native ontology when applicable (ICD10 for diagnoses, CPT or MTHH for procedures, and ATC for medications). For instance, the occurrence of the ICD10 code “H61.23” for the diagnosis of impacted cerumen, bilateral, resulted in two additional parent codes, namely “H61.2” (impacted cerumen) and “H61” (other disorders of external ear).

We used a transformer and GRU as the architectures for our sequence models as they may exhibit different scaling properties and have each demonstrated success in the sequence modeling of medical codes in the EHR^17–19,27,28^. To construct patient representations, sets of codes for each day in the patient timeline were first passed through the embedding bag layer of the networks, which computes the mean code embedding for each day. Next, each mean embedding was concatenated with a vector that captured time information including the patient’s age on that day, the time delta from the previous day, whether that day was the first day of the sequence, and the log transform of the age and time delta. These vectors preserve the varying temporal intervals between patient days and thereby enable CLMBR to fully leverage temporal information^29^. For the transformer architecture, we additionally concatenated two encodings: the first is a standard sine and cosine positional encoding^11^ for the day offset, and the second is the encoding of patient age. Patient representations were then computed by feeding the concatenated vectors into the transformer or GRU, followed by a linear layer with output size equal to the number of dimensions of the patient representation, which was set to 800 in this study.

To predict the set of codes for a given day, *d*_*i*_, the patient representation from the previous day, *d*_*i−1*_, was used. We formulated the set prediction problem as a series of independent binary classification problems, where the probability of a given code was computed via a sigmoid transformation of the dot product between the code embedding and the patient representation. To deal with the computational complexity of the matrix product induced by the large code space, we used a variant of the hierarchical softmax optimization^30^ in which we replaced the softmax transformations with sigmoid transformations. The hierarchical structures of the code space were the same as the ones used for ontology extension (e.g., the hierarchical structure in the ICD10 vocabulary for ICD10 codes). We used the binary cross entropy loss as the loss function during training.

Once the sequence models were trained, we froze their weights and used them to construct representations (the output of the linear layer) for each patient in the task-specific cohort to be used by downstream models for clinical prediction tasks. For hospital mortality, long LOS, and ICU admissions, patient representations were obtained up until the day of admission, whereas for 30-day readmission patient representations were obtained up until the day of discharge.

### Experimental setup

First, we conducted a baseline experiment to establish count-based model performance for each of the four clinical prediction tasks and to investigate whether model performance degraded over time as a result of temporal distribution shift. We trained logistic regression models on count-based representations constructed for patients admitted between 2009 and 2012 (count-LR) and evaluated the models on all years from 2009 to 2021. The years on which the models were trained (2009–2012) constituted the ID years and the subsequent years (2013–2021) constituted the OOD years for the baseline experiment. We also included oracle models that were trained and evaluated on each of the OOD years for comparison.

Next, in Experiment 1, we compared ID and OOD performance of CLMBR-LR to count-LR. For this experiment, ID years were 2009–2012 and the OOD years were 2013–2016 and 2017–2021. To gain insight into relative performance, we subtracted each model’s OOD performance in 2013–2016 and 2017–2021 by its ID performance in 2009–2012 for the same representation construction and modeling approach. As a sensitivity analysis, we trained and compared task-specific models on count-based representations and CLMBR using light gradient-boosted machines (LightGBM) instead of logistic regression.

We additionally conducted analyses in Experiment 1 to examine the contribution of CLMBR pretraining. The first analysis compared CLMBR-LR to end-to-end neural networks (ETE) with the same architecture (that is, GRU and transformer) with the hypothesis that CLMBR-LR should perform as well as or better than its ETE counterpart. The second analysis investigated the Pearson correlation between each CLMBR model’s pretraining performance and the downstream logistic regression performance in each clinical prediction task. Performance for this analysis was measured using binary cross-entropy loss.

**Figure 2:**
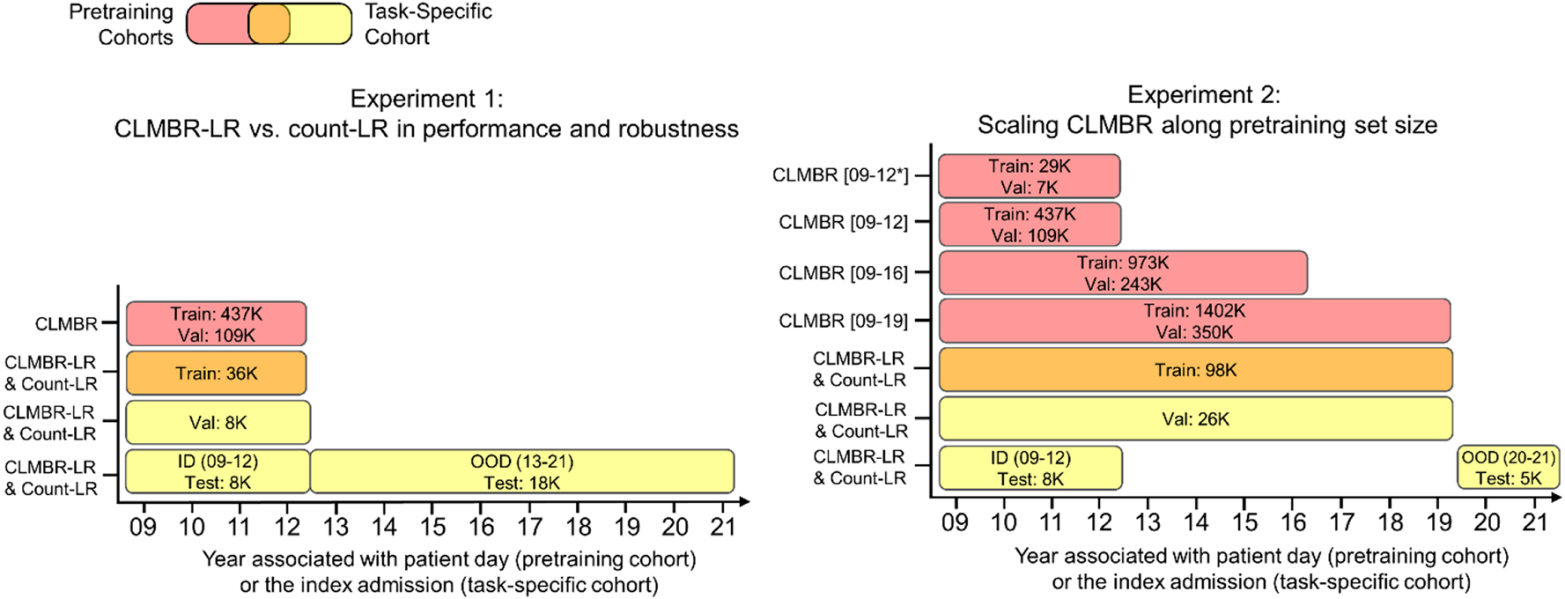
Year range for cohort selection and the dataset size for CLMBR pretraining and task-specific models. The Venn diagram (top left) illustrates the overlap between patients in the pretraining cohorts and the training set of the task-specific cohort. The asterisk in CLMBR [09–12*] indicates a subset of CLMBR [09–12] and only included patients who were admitted to an inpatient unit between 2009 and 2012. Flow diagrams of patient cohort allocation in supplementary Methods online detail the assignment of patients into each cohort and the data splitting procedure. Note that the datasets for the task-specific cohort may vary in the number of patients across clinical outcomes due to additional exclusion of patients for each outcome. For instance, for ICU admission predictions we excluded patients who were transferred on the first day of the index admission since these events would have occurred before prediction time. *CLMBR* Clinical language model-based representation; *LR* Logistic regression; *ID* In-distribution; *OOD* Out-of-distribution.

In Experiment 2, we examined whether performance and robustness of CLMBR would improve with scale and whether the two CLMBR architectures—GRU and transformer—scale differently. While language foundation models have been scaled along various factors such as model size, pretraining set size, and the amount of compute^15,31^, here we focused on scaling pretraining set size, from 36 K to 1.8 M patients. The flow diagrams of patient cohort allocation in Supplementary Methods online and Fig. 2 illustrate patient selection and the number of patients in each pretraining set size. We acknowledge that larger pretraining sets contained data that were also closer in time to the task-specific test sets (for instance the largest pretraining set spanned 2009 to 2019 while the smallest pretraining set spanned 2009 to 2012). The proximity to task-specific test sets may account for some of the differences in performance across pretraining sets. However, both architectures were affected the same way and we were interested in whether there was a difference between the architectures in their performance across these pretraining sets. For this experiment, task-specific logistic regression models were trained on count-based representations and CLMBR constructed for patients admitted between 2009 and 2019 and were evaluated on patients admitted between 2009 and 2012 (ID) and 2020–2021 (OOD).

Finally, to aid clinical interpretation of the changes in performance between count-LR and CLMBR-LR, we quantified the numbers of decisions that would have been affected if CLMBR-LR was used instead of count-LR for tasks in which performance degraded over time. Specifically, we selected the best CLMBR-LR and calculated the proportion of patients that would have been classified correctly and incorrectly with CLMBR-LR instead of count-LR across various risk thresholds.

#### Model development

Figure 2 illustrates the dataset slices for each cohort. We extracted count-based representations and CLMBR for each patient in the task-specific cohort. For count-based representations, we additionally pruned features with less than 25 observations in the training set for each task separately. We then pruned the same features from the validation and test sets. To tune CLMBR, we performed grid search over the hyperparameter settings in Experiment 1 and subsequently used the selected hyperparameter setting for each CLMBR architecture in Experiment 2. The hyperparameters consisted of learning rate, dropout rate, and batch size. Supplementary Methods online detail the hyperparameter grid and the selected hyperparameter settings for transformer-based CLMBR (see Supplementary GRU Experiment online for details on GRU-based CLMBR). For hyperparameter selection, we trained CLMBR on the timelines of 80% of the patients in the pretraining set, and selected hyperparameter settings based on model performance in the left out 20% of the pretraining set. Once pretrained, we constructed CLMBR representations for each patient in the task-specific cohort.

After computing the representations, we trained logistic regression with L2-regularization on count based and CLMBR representations for each clinical outcome in the task-specific training set of 2009–2012 in Experiment 1, and 2009–2019 in Experiment 2. Hyperparameter tuning was done on L2 regularization strength, which ranged from 10^−6^ to 10^2^ in increments of powers of 10. We selected hyperparameter values based on the model’s binary cross entropy loss in the task-specific validation set of the same year group as the training set. Oracle models for each OOD year (2013–2021) as comparisons for count-LR in the baseline experiment were trained on count-based representations in the task-specific training set of the OOD year, and hyperparameters were selected based on performance in the task-specific validation set of the OOD year. Finally, the ETE models for Experiment 1 were trained for each clinical outcome separately on the task-specific training set of 2009–2012, and hyperparameter tuning was conducted using a similar grid as CLMBR. We selected hyperparameter settings for ETE based on model performance in the task-specific validation set of 2009–2012. Supplementary Methods online provide details on the selected hyperparameter setting for logistic regression and ETE for each clinical prediction task.

CLMBR and ETE were implemented using Pytorch^32^ and were trained on a cluster of Nvidia V100 GPUs. We used the Sci-kit Learn’s^33^ implementation of logistic regression. Analyses were implemented in Python 3.8^34^.

#### Model evaluation

We evaluated each model’s discrimination performance in the task-specific test sets using the area-under-the-receiver-operating-characteristic curve (AUROC) and the calibrated area-under-the-precision-recall curve (AUPRC_*C*_)^35^. AUPRC_*C*_ computes the precision using a reference outcome prevalence, here set as the prevalence in the ID year group 2009–2012. Thus, AUPRC_*C*_ is invariant to change in outcome prevalence in OOD years and allows us to better interpret its variation over time. We used the absolute calibration error (ACE)^24^ as a measure of calibration. ACE is similar to the integrated calibration index^36^ but applies a logistic regression estimator to the logit of the predicted probability outputs rather than locally weighted least squares and is thus more computationally efficient. To evaluate the scaling of CLMBR to pretraining set size in Experiment 2, we obtained the slope of the regression line fitted on the performance of each CLMBR architecture along the various pretraining set sizes separately for each performance metric, task, and year group.

#### Statistical analysis

For each metric, we computed the median and 95% confidence interval (CI) of the distribution over performance in the task-specific test set obtained from 1000 bootstrap samples. To compare models, we computed the 95% CI of their differences between a pair of models over 1000 bootstrap samples. Statistical significance was defined as comparisons where the 95% CI did not cross 0.

### Ethics approval and consent to participate

This study used de-identified data and so the requirement for Institutional Review Board approval and participant informed consent were waived by Stanford Medical Center.

## Results

**Figure 3:**
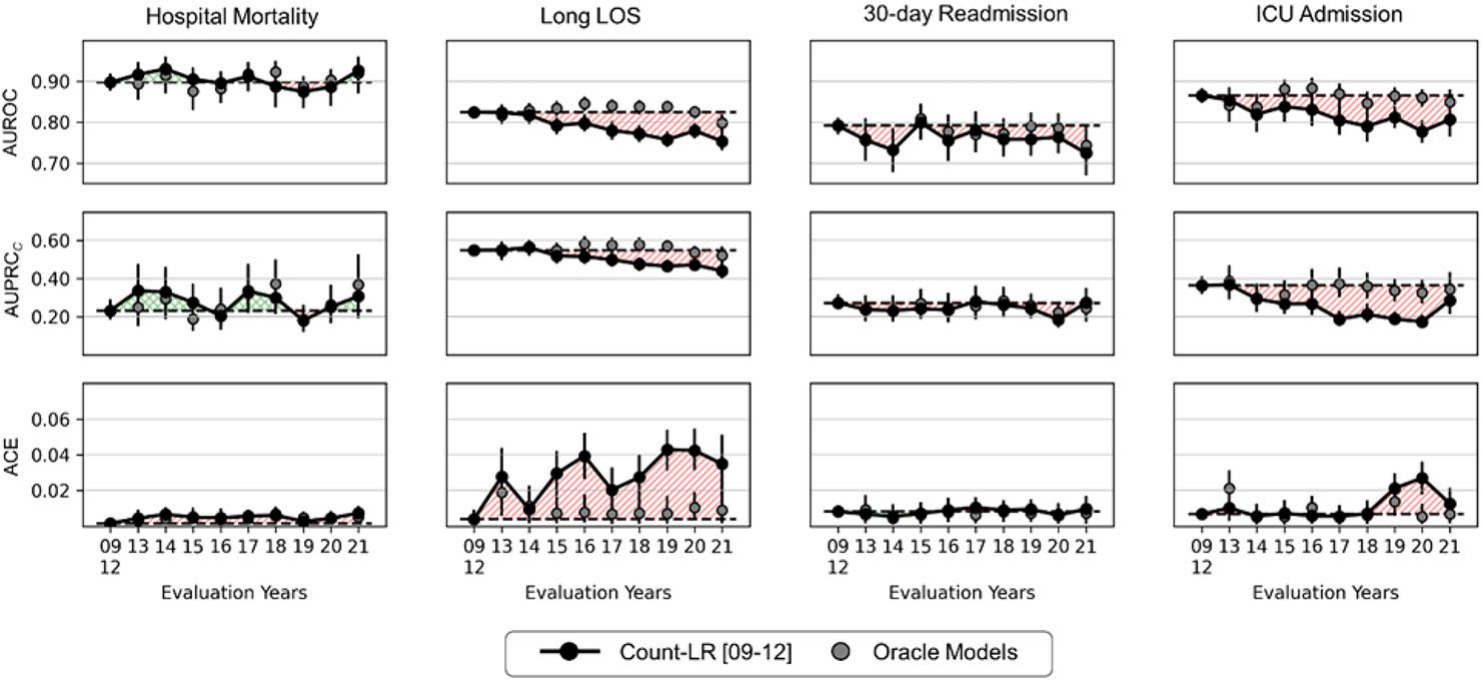
The impact of temporal distribution shift on the performance (AUROC, AUPRC_*C*_, and ACE) of logistic regression models trained on count-based representations (count-LR). Shaded regions indicate time windows in which performance in out-of-distribution years (2013–2021) is worse (red) or better (green) than performance in the in-distribution year group (2009–2012). A Larger red shaded region indicates more degradation relative to the model’s in-distribution performance. Oracle models were trained and evaluated on each of the out-of-distribution years. Error bars indicate 95% confidence interval obtained from 1000 bootstrap iterations. *AUROC* Area under the receiver operating characteristics curve; *AUPRC*_*C*_ Calibrated area under the precision recall curve; *ACE* Absolute calibration error; *LOS* Length of stay; *ICU* Intensive care unit.

Supplementary Table S1 online presents task-specific cohort characteristics for each year and outcome prevalence. Figure 3 shows the impact of temporal distribution shift on performance (AUROC, AUPRC_*C*_, and ACE) of count-LR in the baseline experiment. Model degradation occurred in the OOD years (2013–2021) for long LOS and ICU admission prediction tasks, with larger degradations observed in 2017–2021.

**Figure 4:**
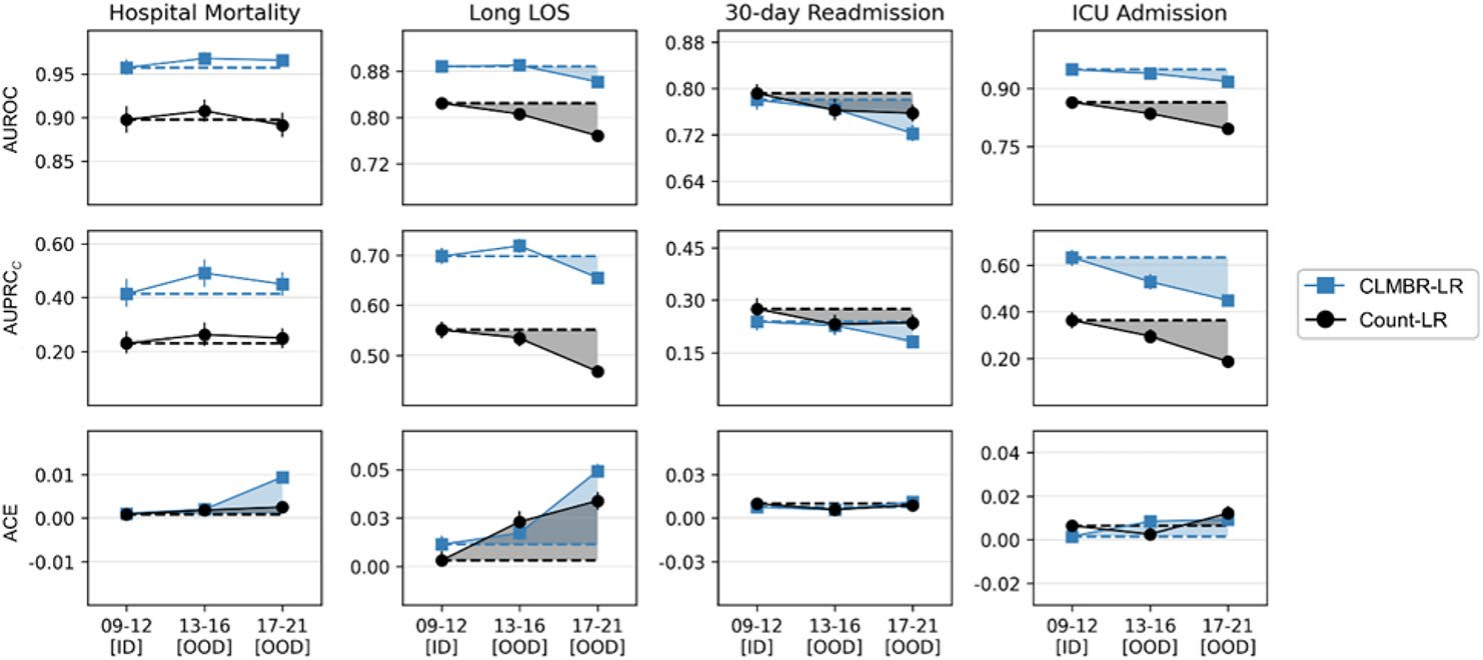
Performance of transformer-based CLMBR-LR and count-LR in the in-distribution (ID) year group and their decay (shaded regions) in out-of-distribution (OOD) year groups. A larger shaded region indicates more performance degradation. GRU-based CLMBR-LR results are available in the Supplementary GRU Experiment online. Error bars indicate 95% confidence interval obtained from 1000 bootstrap iterations. Raw performance scores and change in OOD performance relative to ID are provided in Supplementary Tables S2 and S3 online, respectively. *AUROC* Area under the receiver operating characteristics curve; *AUPRC*_*C*_ Calibrated area under the precision recall curve; *ACE* Absolute calibration error; *LOS* Length of stay; *ICU* Intensive care unit; *CLMBR* Clinical language model-based representation; *LR* Logistic regression.

Figure 4 shows the comparison of ID (2009–2012) and OOD (2013–2016 and 2017–2021) performance between CLMBR-LR and count-LR in Experiment 1 (see Supplementary Tables S2 and S3 online for raw performance scores and relative OOD performance, respectively). For brevity, we display and describe transformer-based CLMBR results. GRU-based CLMBR results are qualitatively similar and can be found in Supplementary GRU Experiment online. First, CLMBR-LR outperformed count-LR in discrimination performance in both ID and OOD year groups across all tasks except for 30-day readmission. In addition, CLMBR-LR displayed less degradation in AUROC (mean decay of 1.3% vs. 4.5% for count-LR) and AUPRC_*C*_ (mean decay of 1.6% vs. 8.9% for count-LR) for long LOS and in AUROC in the 2017–2021 year group for ICU admission(3.3% vs. 8.0% for count-LR), whereas count-LR displayed less degradation in AUROC in the 2017–2021 year group for 30-day readmission (4.4% vs. 7.3% for CLMBR-LR). Calibration results were heterogeneous. Furthermore, the sensitivity analysis where we trained and compared task-specific models on count-based representations and CLMBR using LightGBM instead of logistic regression showed qualitatively similar findings (Supplementary LightGBM Experiment online).

To examine the contribution of CLMBR pretraining, comparison of transformer-based CLMBR-LR and ETE in ID and OOD year groups in Supplementary Figure S1 online shows that CLMBR-LR performed as well as or better than its ETE counterpart in all tasks and metrics except for OOD calibration in 2017–2021 for long LOS and hospital mortality. Supplementary Figure S2 online plots the average binary cross entropy loss of CLMBR sequence models (trained using various hyperparameter settings) in the pretraining validation set against the average binary cross entropy loss of their downstream logistic regression models in each task and year group. The performance of CLMBR models had strong positive correlations (Pearson correlation coefficient) with the ID and OOD performance of their downstream logistic regression models in long LOS and ICU admission, and weak to strong positive correlation with hospital mortality.

**Figure 5:**
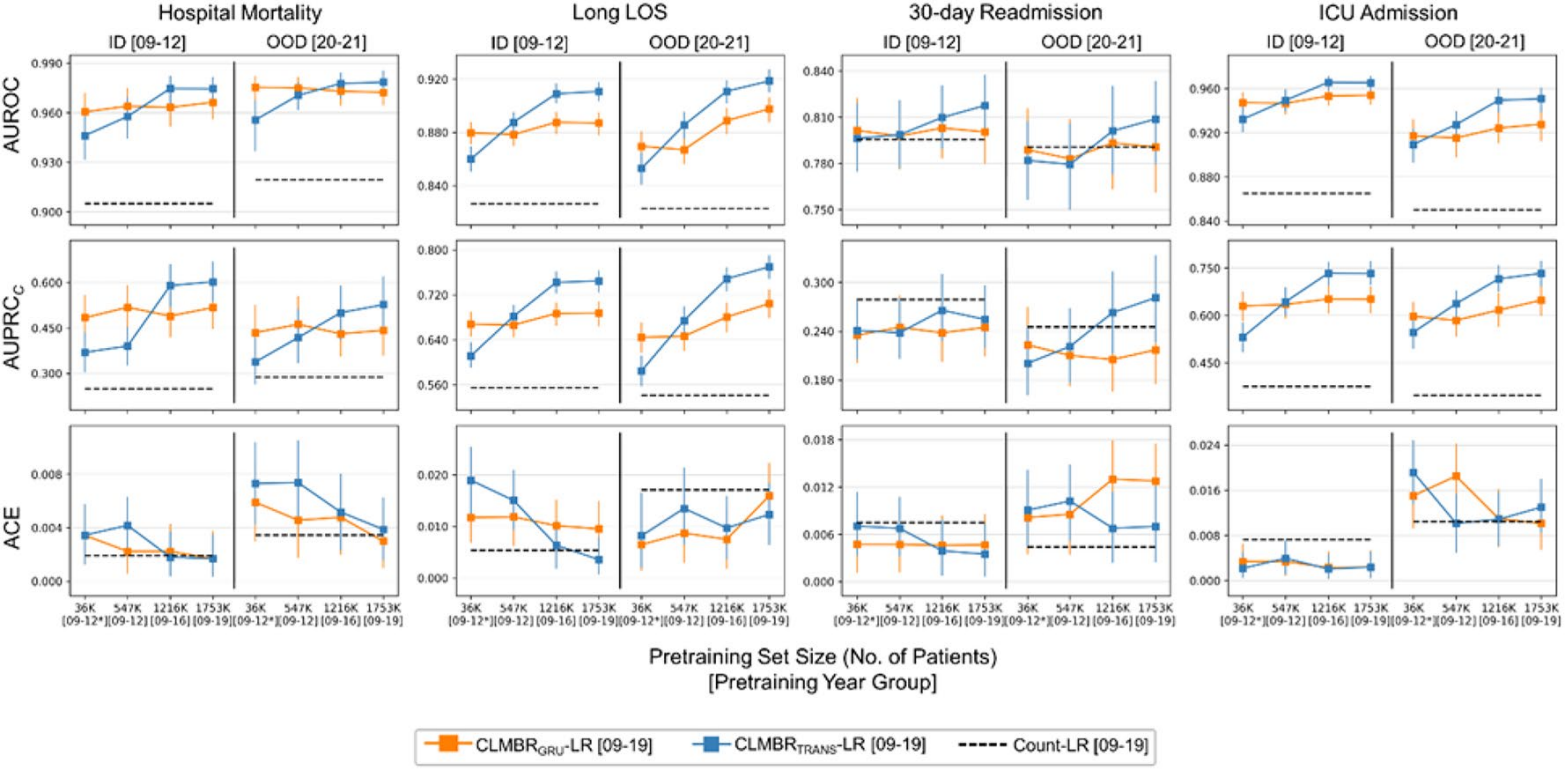
Scaling of GRU- and transformer-based CLMBR along pretraining set size. A more positively sloped curve (negative for ACE) indicates better performance or robustness (performance in the OOD year group) along increasing pretraining set size. Note that the number of patients include both training and validation sets of the pretraining cohort. The asterisk in [09–12*] indicates a subset of patients in [09–12] that have been admitted to an inpatient unit during the year range. Raw performance scores and slopes are provided in Supplementary Tables S6 and S7 online, respectively. *AUROC* Area under the receiver operating characteristics curve; *AUPRC*_*C*_ Calibrated area under the precision recall curve; *ACE* absolute calibration error; *LR* Logistic regression; *CLMBR* Clinical language model-based representations; *GRU* Gradient recurrent unit; *TRANS* Transformer; *LOS* Length of stay; *ICU* Intensive care unit.

Figure 5 shows the ID and OOD performance of GRU-based and transformer-based CLMBR as a function of pretraining set size (see Supplementary Tables S6 and S7 online for raw performance scores and slopes, respectively). Generally, GRU-based CLMBR performed better with smaller pretraining set sizes whereas transformer-based CLMBR exhibited better scaling of discrimination performance to pretraining set size, outperforming GRU-based CLMBR at larger pretraining set sizes. Scaling of calibration was heterogeneous.

**Figure 6:**
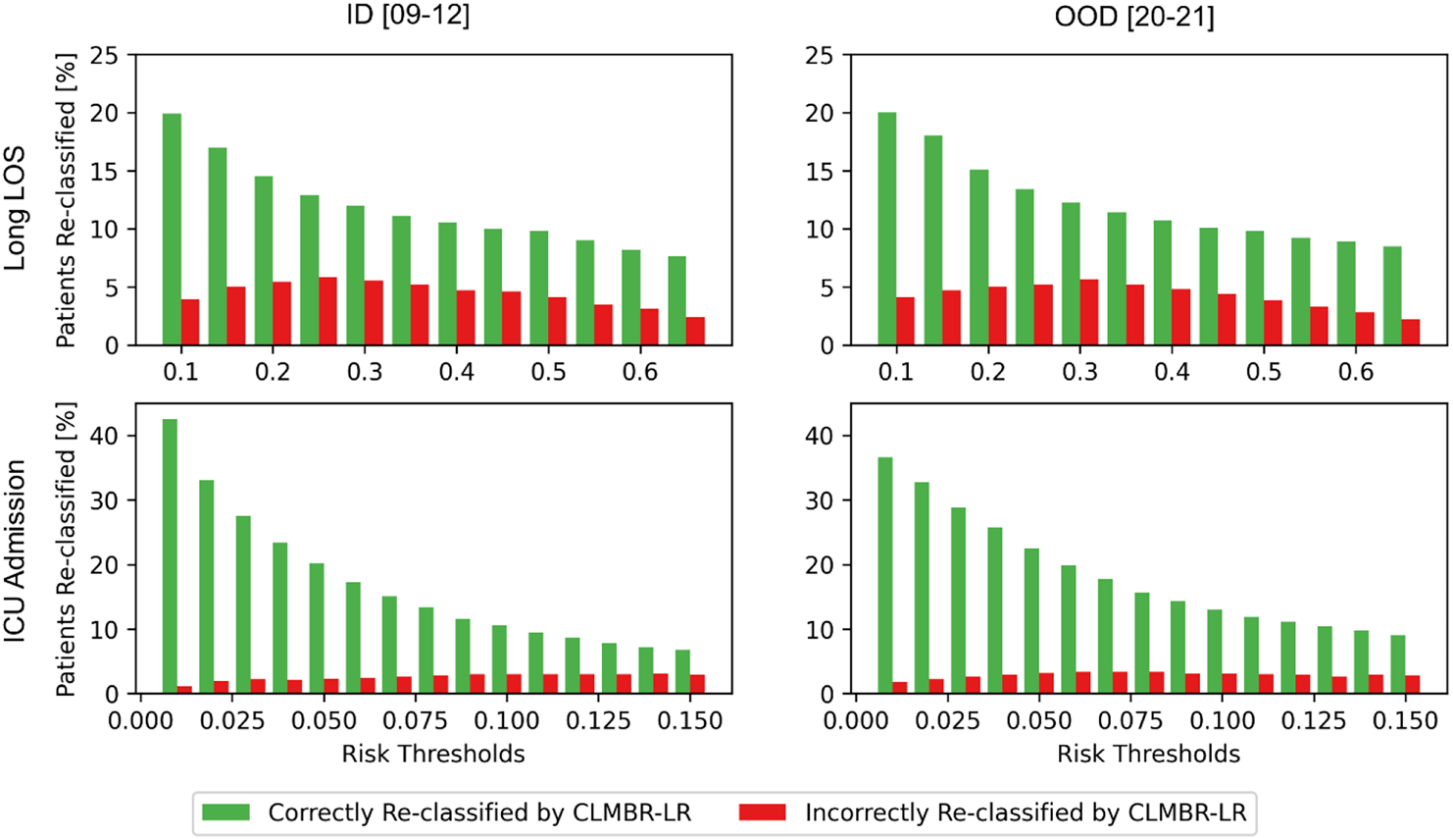
The proportion of patients correctly and incorrectly re-classified by CLMBR-LR. Green bars indicate the proportion of patients that were incorrectly classified by count-LR but were correctly re-classified by CLMBR-LR. Red bars indicate the proportion of patients that were correctly classified by count-LR but were incorrectly re-classified by CLMBR-LR. Together, these represent the percentage of decisions that would have been affected if CLMBR-LR were used instead of count-LR. *LR* logistic regression; *CLMBR* clinical language model-based representations; *LOS* length of stay; *ICU* intensive care unit.

Figure 6 plots the proportion of patients re-classified differently using CLMBR-LR instead of count-LR in long LOS and ICU admission. For this analysis, we selected the transformer-based CLMBR pretrained on the 2009–2019 pretraining cohort, and logistic regression models were trained on admissions from 2009 to 2019. There were more correct re-classifications than incorrect re-classifications across risk thresholds and year groups for long LOS and ICU admission.

## Discussion

We observed count-LR models resulted in large performance degradation over time for some tasks, namely long LOS and ICU admission. CLMBR-LR generally displayed better discrimination than count-LR in ID and OOD year groups (but could result in worse calibration) and often exhibited less decay in tasks where there is observable degradation of discrimination performance. In addition, whereas GRU-based CLMBR generally performed better with a smaller pretraining set size, the discrimination performance and robustness of transformer-based CLMBR improved more as pretraining set size increased, surpassing GRU-based CLMBR.

Large-scale self-supervised pretraining takes place less frequently and centralizes as well as standardizes model training and feature generation compared to the training of end-to-end models from scratch. Consequently, machine learning practitioners are able to focus on rapid adaptation of foundation models to downstream tasks. This research paper contributes more evidence that this approach brings not only performance benefits over traditional count-based models, but robustness benefits in the presence of temporal distribution shifts. These benefits decrease the need for model retraining and preserves the clinical utility of models deployed into practice. Whether the potential for worse calibration is clinically meaningful will depend on the specific use case.

Developing intrinsic measures of upstream model quality (e.g., measuring language modeling perplexity) that reliably correlate with downstream task accuracy is of great interest and utility to the machine learning community. In NLP, recent work has found poor correlation between perplexity and downstream task accuracy^37^. However, in our setting, we observed strong correlations between performance of the upstream language modeling objective (measured using log loss) and the performance of classification heads in some tasks, but not all. This suggests that the favorable ID and OOD performance are partly attributable to the self-supervised pretraining. We hypothesize that code-based language modeling, unlike in NLP, often directly maps to downstream clinical prediction tasks, since codes may denote a clinical condition of interest. However, many other clinical tasks are highly compositional, meaning they do not map to a clear set of codes, making them harder to capture directly in pretraining. This should be explored in future work alongside other pretraining objectives and intrinsic measures of EHR foundation model performance.

The finding that the transformer-based CLMBR is more scalable whereas GRU-based CLMBR is more sample-efficient is consistent with the idea that models that integrate strong inductive biases like the GRU are more adept with smaller sample sizes, whereas weaker inductive biases of the transformer enable better scaling to larger volumes of data. The latter motivates additional research on learning transformer-based foundation models on larger-scale data as well as multiple modalities to explore and expand their range of capabilities in clinical medicine. In addition, scaling along other factors such as model size and the amount of compute^15,31^ are needed to better understand the scaling laws of foundation models in clinical medicine.

Various EHR foundation models were developed in recent years based on the transformer architecture and pretraining tasks used in BERT. These include, for example, Med-BERT^18^, BEHRT^19^, G-BERT^28^, and CEHR-BERT^29^. These models share similarities with CLMBR in that they are pretrained on EHR-derived patient timelines but differ in the type of medical codes and their vocabulary for inputs; pretraining set size and objective; and the adaptation (or transfer learning) approach. Notably, BERT-based models were adapted by fine-tuning all weights for each downstream task. In contrast, CLMBR was adapted by freezing the weights and learning just a classification head for each clinical task. Our adaptation approach is conservative and less resource-intensive, which enables rapid development of task-specific models. In addition, it has been demonstrated that end-to-end fine-tuning does not always improve performance, particularly in OOD settings^38^. In addition, very little has been published that examines the robustness of an EHR foundation model and its scalability in the presence of temporal distribution shift. This work also compares end-to-end models like LR and GRUs to foundation models (of which CLMBR is just one example).

We postulate that the observed improvement in performance and robustness in CLMBR-LR are enabled in part by the pretraining objective and the size and diversity^12,39^ of the pretraining set. During pretraining, CLMBR learns a representation that captures relationships between raw features (i.e., medical codes). Such a representation is more holistic and is arguably a more time-invariant “view” of the patient. The relationship between this “view” and an outcome (e.g., long length of stay) might therefore be less spurious and more robust to distribution shifts compared to the relationship between raw features and the outcome (learned by end-to-end models like count-LR).

We examined two attributes of OOD performance, namely absolute performance and relative (to ID) performance. It is notable that while CLMBR resulted in generally better absolute discrimination than count-LR, improvement in relative discrimination was more modest. It is likely that absolute performance is more meaningful to clinicians since better relative performance does not necessarily indicate better absolute performance^40^. However, decision makers may be more concerned about using a model that does not perform as well as that originally promised (relative performance). It is for that reason that we choose to report both aspects. In practice, the foundation model can be retrained, fine-tuned, or updated over time via monitoring and updating policies^41^ to mitigate the impact of temporal distribution shift. In addition, structure and certain types of invariances could be incorporated at various stages using metadata^42^, regularization^43^, or contrastive learning^44^.

The strengths of this study include the evaluation of a novel approach to self-supervised representation learning on electronic health records, namely CLMBR, in both ID and OOD settings. Another strength is the adoption of interpretable metrics to evaluate the clinical impact of using CLMBR-based models instead of models trained on count-based representations. However, this study is limited in several ways. First, we only used a single dataset with a limited number of outcomes. Performance of CLMBR may differ in other settings and with other tasks. Second, this work focused on the evaluation of a single foundation modelling approach. Future work should explore other approaches. In addition, our evaluation was restricted to temporal robustness and we did not explore other properties that may have emerged from pretraining at larger scales. Finally, we lack insight into how well CLMBR scales along other factors such as model size and the amount of compute, the effect of various adaptation approaches such as end-to-end fine-tuning of CLMBR weights on the robustness of task-specific models, and the scenarios in which CLMBR may be more or less helpful.

In conclusion, models trained on CLMBR were better than count-LR in discrimination performance and often exhibited less decay in cases where OOD discrimination performance degraded. In addition, performance and robustness of transformer-based CLMBR improved further when more data became available for pretraining. These results suggest that pretraining EHR foundation models at scale is a useful approach for developing clinical prediction models that perform well ID as well as OOD.

## Supplementary Information

Supplementary Information.

## Data Availability

The de-identified Stanford Medicine Research Data Repository OMOP common data model is available through Nero, the highly secure data science platform maintained by Research IT at Stanford Medicine and Stanford Research Computing Center. Access to Nero requires affiliation with a Stanford Principal Investigator and a Stanford identity. However, data are available from the corresponding author upon reasonable request. The code for all analyses is open-source and available at https://github.com/som-shahlab/temp_ds_shift_robustness.
