# Supplementary material for: EHR foundation models improve robustness in the presence of temporal distribution shift
Source: Sci Rep. 2023 Mar 7;13:3767. doi: 10.1038/s41598-023-30820-8 (PMC9992466; doi:10.1038/s41598-023-30820-8)
Supplement: Supplementary file 1 — Supplementary Information. [file 41598_2023_30820_MOESM1_ESM.docx]

**Supplementary Content**

| Pages |  |  |
| --- | --- | --- |
| 2-7 | **Supplementary Methods** | Additional details on cohort selection and model hyperparameters |
| 8 | **Supplementary Table S1** | Task-specific cohort characteristics by year and outcome prevalence |
| 9 | **Supplementary Table S2** | AUROC, AUPRC*_C_*, and ACE for CLMBR-LR and Count-LR in ID and OOD year groups |
| 10 | **Supplementary Table S3** | Change in OOD AUROC, AUPRC*_C_*, and ACE for count-LR and CLMBR-LR relative to ID |
| 11 | **Supplementary Figure S1** | Performance of CLMBR-LR vs. end-to-end transformer in ID and OOD year groups. |
| 12 | **Supplementary Table S4** | AUROC, AUPRC*_C_*, and ACE for CLMBR-LR and end-to-end transformer in ID and OOD year groups |
| 13 | **Supplementary Table S5** | Change in OOD AUROC, AUPRC*_C_*, and ACE for count-LR and end-to-end transformer relative to ID |
| 14 | **Supplementary Figure S2** | Correlation between CLMBR pretraining performance and the performance of downstream logistic regression models |
| 15 | **Supplementary Table S6** | Performance of GRU- and transformer-based CLMBR along increasing pretraining set size |
| 16 | **Supplementary Table S7** | Scaling of GRU- and transformer-based CLMBR to pretraining set size |
| 17-25 | **Supplementary GRU Experiment** | Ablation of CLMBR architecture – replacement of transformer with GRU |
| 26-31 | **Supplementary LightGBM Experiment** | Ablation of classification head – replacement of logistic regression with LightGBM |

**Supplementary Methods.** Additional details on clinical outcomes, feature extraction, models and learning algorithms.

1. Flow diagram of patient cohort allocation with respect to pretraining and task-specific cohorts for experiment 1
2. Flow diagram of patient cohort allocation with respect to pretraining and task-specific cohorts for experiment 2
3. CLMBR hyperparameters
4. Selected hyperparameter settings for CLMBR and end-to-end models
5. Selected hyperparameter setting for logistic regression models

**Supplementary Methods I.** Flow diagram of patient cohort allocation with respect to pretraining and task-specific cohorts for experiment 1 - CLMBR-LR vs. count-LR in performance and robustness. The diagram illustrates the inclusion and exclusion criteria for the assignment of patients to CLMBR pretraining and task-specific cohorts as well as the splitting of patients into training, validation, and test sets.


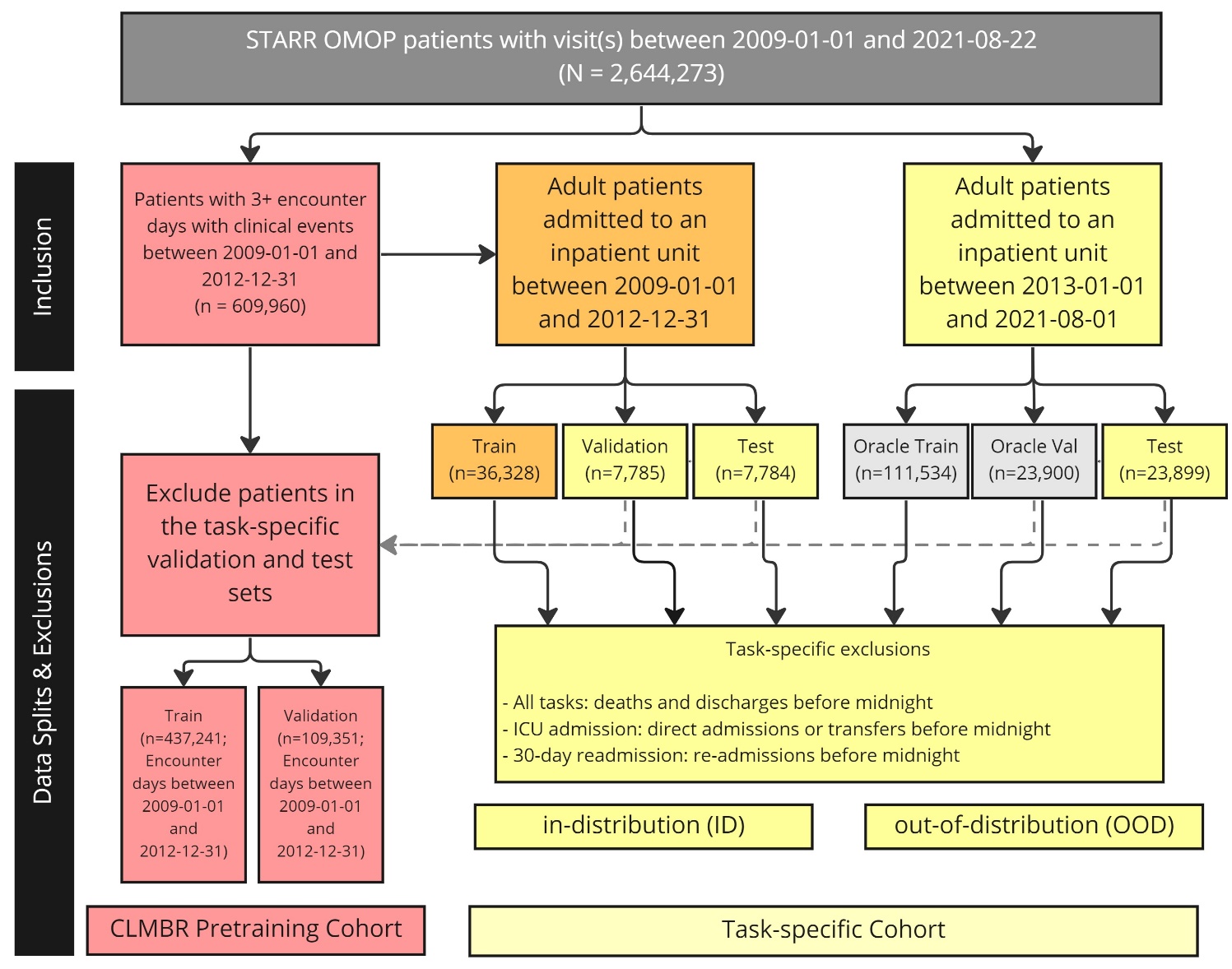


**Supplementary Methods II.** Flow diagram of patient cohort allocation with respect to pretraining and task-specific cohorts for experiment 2 – Scaling CLMBR along pretraining set size. The diagram illustrates the inclusion and exclusion criteria for the assignment of patients to CLMBR pretraining and task-specific cohorts as well as the splitting of patients into training, validation, and test sets.


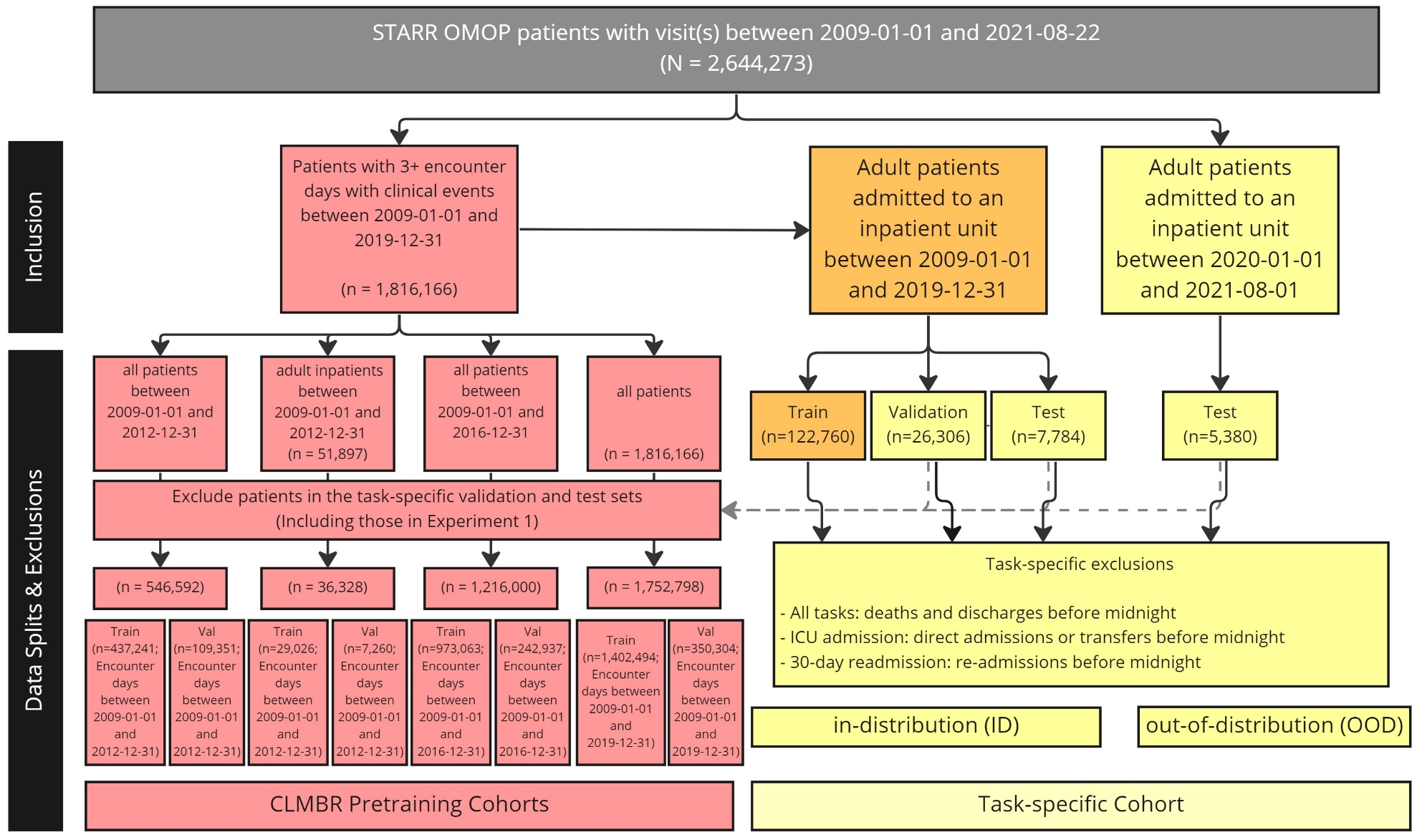


**Supplementary Methods III.** Transformer hyperparameter grid

| **Architecture** | **Task** | **Hyperparameter** | **Values** |
| --- | --- | --- | --- |
| Transformer | Sequence Modeling & End-to-end | Learning rate | 0.0001, 0.00001 |
| Transformer | Sequence Modeling & End-to-end | Dropout | 0, 0.2, 0.4 |
| Transformer | Sequence Modeling | Batch size | 2000, 4000 |
| Transformer | End-to-end | Code dropout | 0.2, 0.4 |
| Transformer | End-to-end | Transformer Layers^a^ | 6, 12 |

CLMBR models were pretrained up to 10,000 epochs and stopped early if no improvement in the validation set was observed for 100 epochs. End-to-end models were trained for 50 epochs.

^a^ Each layer is consisted of a multi-head attention (number of heads = 8) layer and two feed-forward layers. The number of transformer layers is fixed at 6 for CLMBR.

Abbreviations: CLMBR: clinical language model-based representations.

**Supplementary Methods IV**. Selected transformer hyperparameter values

| **Architecture** | **Task** | **Hyperparameter** | **Value** |
| --- | --- | --- | --- |
| Transformer | Sequence modeling (CLMBR pretraining) | Learning rate | 0.0001 |
| Transformer | Sequence modeling  (CLMBR pretraining) | Batch size | 2000 |
| Transformer | Sequence modeling  (CLMBR pretraining) | Dropout | 0.4 |
| Transformer | Hospital Mortality | Learning rate | 0.0001 |
| Transformer | Hospital Mortality | Dropout | 0.4 |
| Transformer | Hospital Mortality | Code Dropout | 0.2 |
| Transformer | Hospital Mortality | Transformer Layers | 6 |
| Transformer | Long LOS | Learning rate | 0.0001 |
| Transformer | Long LOS | Dropout | 0.2 |
| Transformer | Long LOS | Code Dropout | 0.4 |
| Transformer | Long LOS | Transformer Layers | 6 |
| Transformer | ICU Admission | Learning rate | 0.0001 |
| Transformer | ICU Admission | Dropout | 0 |
| Transformer | ICU Admission | Code Dropout | 0.4 |
| Transformer | ICU Admission | Transformer Layers | 6 |
| Transformer | 30d Readmission | Learning rate | 0.0001 |
| Transformer | 30d Readmission | Dropout | 0 |
| Transformer | 30d Readmission | Code Dropout | 0.4 |
| Transformer | 30d Readmission | Transformer Layers | 6 |

Abbreviations: CLMBR: clinical language model based representations

**Supplementary Methods V**. Selected logistic regression hyperparameter values

| **Featurization** | **Task** | **L2 Regularization Strength** |
| --- | --- | --- |
| CLMBR (Transformer) | Hospital Mortality | 0.001 |
| CLMBR (Transformer) | Long LOS | 0.001 |
| CLMBR (Transformer) | ICU Admission | 0.001 |
| CLMBR (Transformer) | 30d readmission | 0.0001 |
| Count-based | Hospital Mortality | 0.01 |
| Count-based | Long LOS | 0.01 |
| Count-based | ICU Admission | 0.01 |
| Count-based | 30d readmission | 0.001 |

^a^Smaller values indicate stronger regularization. Search was conducted over values ranging from 10^-6^ to 10^2^ in powers of 10

Abbreviations: CLMBR: clinical language model-based representations; ICU: intensive care unit; LOS: length of stay

**Supplementary Table S1.** Task-specific cohort characteristics by year

|  | **Year** | | | | | | | | | | | | |
| --- | --- | --- | --- | --- | --- | --- | --- | --- | --- | --- | --- | --- | --- |
|  | **2009** | **2010** | **2011** | **2012** | **2013** | **2014** | **2015** | **2016** | **2017** | **2018** | **2019** | **2020** | **2021** |
| **No.** | 12727 | 12930 | 13104 | 13136 | 13117 | 15235 | 17241 | 16980 | 17198 | 21187 | 22514 | 22352 | 13509 |
| **Mean age in years ± SD** | 57±18 | 57±18 | 57±18 | 57±18 | 57±18 | 53±19 | 52±19 | 52±19 | 53±19 | 53±20 | 53±20 | 53±20 | 54±20 |
| **Sex, No. (%)** | | | | | | | | | | | | | |
| **Female** | 6572 (52%) | 6579 (51%) | 6767 (52%) | 6576 (50%) | 6668 (51%) | 8820 (58%) | 10483 (61%) | 10310 (61%) | 10269 (60%) | 12874 (61%) | 13556 (60%) | 13234 (59%) | 8010 (59%) |
| **Male** | 6154 (48%) | 6351 (49%) | 6337 (48%) | 6559 (50%) | 6448 (49%) | 6414 (42%) | 6757 (39%) | 6670 (39%) | 6929 (40%) | 8313 (39%) | 8956 (40%) | 9116 (41%) | 5496 (41%) |
| **Race, No. (%)** | | | | | | | | | | | | | |
| **White** | 7054 (55%) | 8106 (63%) | 8091 (62%) | 7848 (60%) | 7585 (58%) | 8092 (53%) | 8620 (50%) | 8174 (48%) | 8328 (48%) | 10164 (48%) | 10866 (48%) | 10101 (45%) | 5908 (44%) |
| **Other** | 5673 (45%) | 4824 (37%) | 5013 (38%) | 5288 (40%) | 5532 (42%) | 7143 (47%) | 8621 (50%) | 8806 (52%) | 8870 (52%) | 11023 (52%) | 11648 (52%) | 12251 (55%) | 7601 (56%) |
| **Clinical Outcome, No. (%)** | | | | | | | | | | | | | |
| **In-Hospital Mortality** | 267 (2%) | 317 (2%) | 337 (3%) | 337 (3%) | 340 (3%) | 321 (2%) | 345 (2%) | 345 (2%) | 335 (2%) | 402 (2%) | 379 (2%) | 459 (2%) | 249 (2%) |
| **LOS >7 Days** | 2680 (21%) | 2718 (21%) | 2719 (21%) | 2739 (21%) | 2703 (21%) | 2939 (19%) | 3065 (18%) | 3364 (20%) | 3263 (19%) | 3767 (18%) | 4078 (18%) | 4300 (19%) | 2709 (20%) |
| **30-Day Readmission** | 658 (5%) | 760 (6%) | 706 (6%) | 681 (5%) | 687 (5%) | 780 (5%) | 813 (5%) | 767 (5%) | 762 (5%) | 1012 (5%) | 1079 (5%) | 1080 (5%) | 619 (5%) |
| **ICU Admission** | 314 (2%) | 788 (6%) | 782 (6%) | 789 (6%) | 835 (6%) | 928 (6%) | 926 (5%) | 953 (6%) | 910 (5%) | 1180 (6%) | 1302 (6%) | 1600 (7%) | 818 (6%) |

Abbreviations. SD: standard deviation; LOS: long length of stay; ICU: intensive care unit.

**Supplementary Table S2.** AUROC, AUPRC*_C_*, and ACE for count-LR and CLMBR-LR in 2009-2012 (ID), 2013-2016 (OOD), and 2017-2021 (OOD).

| **Task** | **Metric** | **Model** | **2009-2012** | **2013-2016** | **2017-2021** |
| --- | --- | --- | --- | --- | --- |
| Hospital Mortality | AUROC | Count-LR | 0.898 | 0.908 | 0.891 |
|  |  | CLMBR-LR | **0.957** | **0.968** | **0.966** |
|  | AUPRC*_C_* | Count-LR | 0.23 | 0.261 | 0.248 |
|  |  | CLMBR-LR | **0.413** | **0.491** | **0.45** |
|  | ACE | Count-LR | 0.001 | 0.003 | **0.004** |
|  |  | CLMBR-LR | 0.002 | 0.003 | 0.014 |
| Long LOS | AUROC | Count-LR | 0.825 | 0.806 | 0.769 |
|  |  | CLMBR-LR | **0.888** | **0.89** | **0.861** |
|  | AUPRC*_C_* | Count-LR | 0.55 | 0.534 | 0.468 |
|  |  | CLMBR-LR | **0.698** | **0.719** | **0.655** |
|  | ACE | Count-LR | **0.003** | 0.023 | **0.034** |
|  |  | CLMBR-LR | 0.011 | **0.017** | 0.049 |
| 30-day readmission | AUROC | Count-LR | 0.792 | 0.762 | **0.757** |
|  |  | CLMBR-LR | 0.78 | 0.765 | 0.723 |
|  | AUPRC*_C_* | Count-LR | **0.275** | 0.23 | **0.235** |
|  |  | CLMBR-LR | 0.238 | 0.227 | 0.182 |
|  | ACE | Count-LR | 0.008 | 0.005 | 0.007 |
|  |  | CLMBR-LR | 0.006 | 0.005 | 0.009 |
| ICU admission | AUROC | Count-LR | 0.865 | 0.835 | 0.796 |
|  |  | CLMBR-LR | **0.949** | **0.939** | **0.918** |
|  | AUPRC*_C_* | Count-LR | 0.363 | 0.295 | 0.187 |
|  |  | CLMBR-LR | **0.633** | **0.529** | **0.449** |
|  | ACE | Count-LR | 0.006 | **0.003** | 0.012 |
|  |  | CLMBR-LR | **0.002** | 0.008 | 0.009 |

Bolded values indicate the significantly better performing model between Count-LR and CLMBR-LR. Significance is determined based on 95% confidence intervals calculated from the distribution of differences obtained over 1000 iterations.

Abbreviations: AUROC: area under the receiver operating characteristics curve; AUPRC*_C_*: calibrated area under the precision recall curve; ACE: absolute calibration error; CLMBR: clinical language model-based representation; LR: logistic regression; LOS: length of stay; ICU: intensive care unit; ID: in-distribution; OOD: out-of-distribution.

**Supplementary Table S3.** Change in OOD AUROC, AUPRC*_C_*, and ACE for count-LR and CLMBR-LR relative to ID

| **Task** | **Metric** | **Model** | **2013-2016** | **2017-2021** |
| --- | --- | --- | --- | --- |
| Hospital Mortality | Δ-AUROC | Count-LR | 0.01 | -0.006 |
|  |  | CLMBR-LR | 0.011 | 0.008 |
|  | Δ- AUPRC*_C_* | Count-LR | 0.031 | 0.018 |
|  |  | CLMBR-LR | 0.078 | 0.036 |
|  | Δ-ACE | Count-LR | 0.001 | **0.002** |
|  |  | CLMBR-LR | 0.001 | 0.013 |
| Long LOS | Δ-AUROC | Count-LR | -0.019 | -0.056 |
|  |  | CLMBR-LR | **0.002** | **-0.026** |
|  | Δ- AUPRC*_C_* | Count-LR | -0.016 | -0.082 |
|  |  | CLMBR-LR | **0.021** | **-0.043** |
|  | Δ-ACE | Count-LR | 0.02 | **0.031** |
|  |  | CLMBR-LR | **0.006** | 0.038 |
| 30-day readmission | Δ-AUROC | Count-LR | -0.029 | **-0.034** |
|  |  | CLMBR-LR | -0.016 | -0.058 |
|  | Δ- AUPRC*_C_* | Count-LR | -0.044 | -0.039 |
|  |  | CLMBR-LR | -0.011 | -0.057 |
|  | Δ-ACE | Count-LR | -0.003 | -0.001 |
|  |  | CLMBR-LR | -0.002 | 0.003 |
| ICU admission | Δ-AUROC | Count-LR | -0.029 | -0.069 |
|  |  | CLMBR-LR | -0.01 | **-0.031** |
|  | Δ- AUPRC*_C_* | Count-LR | -0.068 | -0.176 |
|  |  | CLMBR-LR | -0.104 | -0.184 |
|  | Δ-ACE | Count-LR | **-0.004** | 0.006 |
|  |  | CLMBR-LR | 0.007 | 0.008 |

Deltas were obtained by subtracting the model’s OOD performance by its in-distribution performance. Bolded values indicate the significantly more robust model between Count-LR and CLMBR-LR. Significance is determined based on 95% confidence intervals calculated from the distribution of differences obtained over 1000 iterations.

Abbreviations: AUROC: area under the receiver operating characteristics curve; AUPRC*_C_*: calibrated area under the precision recall curve; ACE: absolute calibration error; CLMBR: clinical language model-based representation; LR: logistic regression; LOS: length of stay; ICU: intensive care unit; ID: in-distribution; OOD: out-of-distribution.

**Supplementary Figure S1**. Performance (AUROC, AUPRC*_C_*, and ACE) of transformer-based CLMBR-LR vs. end-to-end transformer (ETE) in 2009-2012 (09-12), 2013-2016 (13-16), and 2017-2021 (17-21). CLMBR-LR performed as well as or better than ETE with a few exceptions in OOD calibration. The shaded region for each model indicates performance degradation in OOD year groups relative to the ID year group. A larger shaded region indicates more degradation of performance. Error bars indicate 95% confidence interval obtained from 1000 bootstrap iterations. Raw performance values and their change in OOD relative to ID year groups are provided in Supplementary Tables S4 and S5, respectively.

Abbreviations: AUROC: area under the receiver operating characteristics curve; AUPRC*_C_*: calibrated area under the precision recall curve; ACE: absolute calibration error; LOS: length of stay; ICU: intensive care unit; CLMBR: clinical language model-based representation; LR: logistic regression. TRANS: transformer


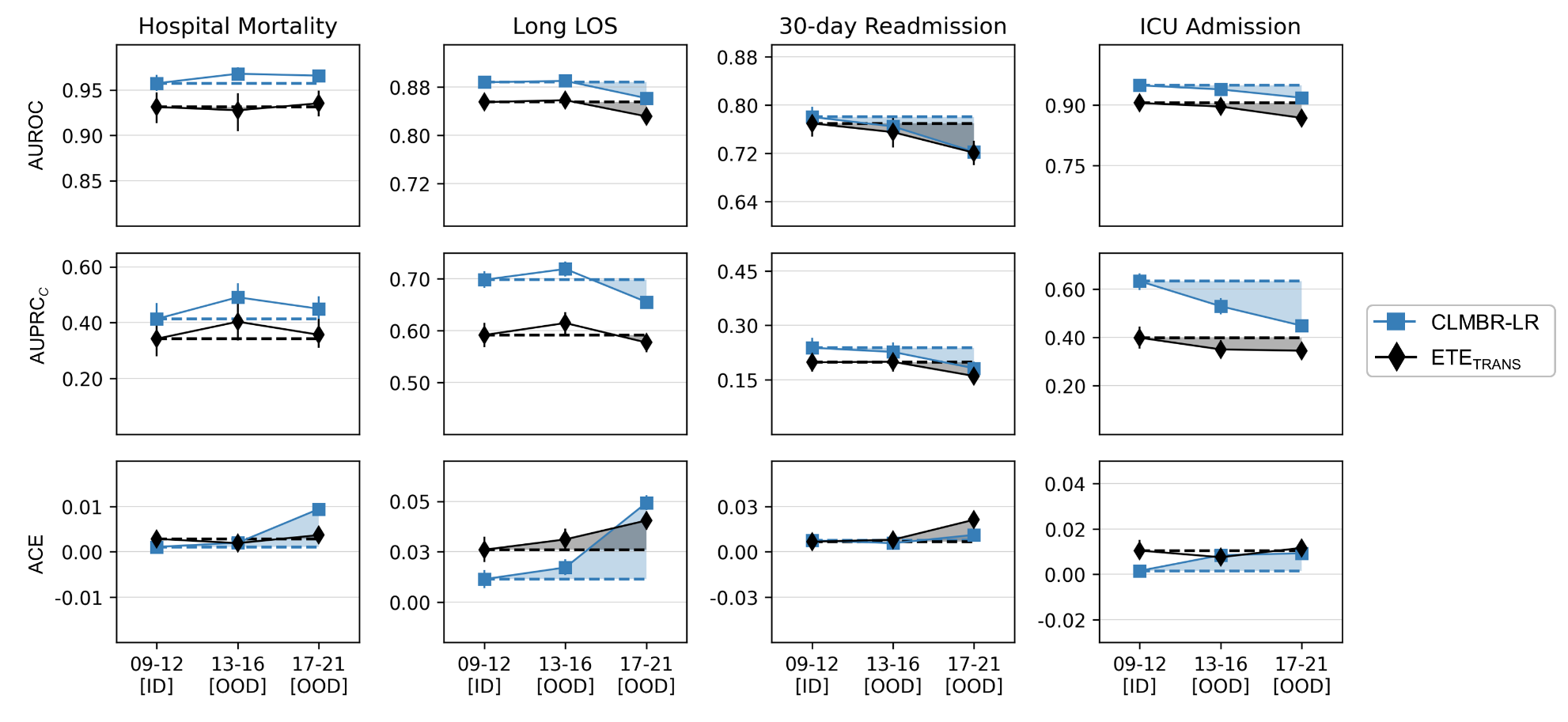


**Supplementary Table S4.** AUROC, AUPRC*_C_*, and ACE for CLMBR-LR and end-to-end transformer (ETE) in 2009-2012 (ID), 2013-2016 (OOD), and 2017-2021 (OOD).

| **Task** | **Metric** | **Model** | **2009-2012** | **2013-2016** | **2017-2021** |
| --- | --- | --- | --- | --- | --- |
| Hospital Mortality | AUROC | ETE | 0.931 | 0.928 | 0.935 |
|  |  | CLMBR-LR | **0.957** | **0.968** | **0.966** |
|  | AUPRC*_C_* | ETE | 0.342 | 0.403 | 0.356 |
|  |  | CLMBR-LR | **0.413** | **0.491** | **0.45** |
|  | ACE | ETE | 0.004 | 0.003 | **0.006** |
|  |  | CLMBR-LR | **0.002** | 0.003 | 0.014 |
| Long LOS | AUROC | ETE | 0.855 | 0.858 | 0.831 |
|  |  | CLMBR-LR | **0.888** | **0.89** | **0.861** |
|  | AUPRC*_C_* | ETE | 0.591 | 0.615 | 0.577 |
|  |  | CLMBR-LR | **0.698** | **0.719** | **0.655** |
|  | ACE | ETE | 0.026 | 0.031 | **0.041** |
|  |  | CLMBR-LR | **0.011** | **0.017** | 0.049 |
| 30-day readmission | AUROC | ETE | 0.77 | 0.755 | 0.721 |
|  |  | CLMBR-LR | 0.78 | 0.765 | 0.723 |
|  | AUPRC*_C_* | ETE | 0.198 | 0.2 | 0.16 |
|  |  | CLMBR-LR | **0.238** | **0.227** | **0.182** |
|  | ACE | ETE | 0.006 | 0.007 | 0.018 |
|  |  | CLMBR-LR | 0.006 | 0.005 | **0.009** |
| ICU admission | AUROC | ETE | 0.905 | 0.896 | 0.868 |
|  |  | CLMBR-LR | **0.949** | **0.939** | **0.918** |
|  | AUPRC*_C_* | ETE | 0.399 | 0.351 | 0.345 |
|  |  | CLMBR-LR | **0.633** | **0.529** | **0.449** |
|  | ACE | ETE | 0.011 | 0.008 | 0.012 |
|  |  | CLMBR-LR | **0.002** | 0.008 | 0.009 |

Bolded values indicate the significantly better performing model between ETE and CLMBR-LR. Significance is determined based on 95% confidence intervals calculated from the distribution of differences obtained over 1000 iterations.

Abbreviations: AUROC: area under the receiver operating characteristics curve; AUPRC*_C_*: calibrated area under the precision recall curve; ACE: absolute calibration error; CLMBR: clinical language model-based representation; LR: logistic regression; LOS: length of stay; ICU: intensive care unit; ID: in-distribution; OOD: out-of-distribution.

**Supplementary Table S5.** Change in OOD AUROC, AUPRC*_C_*, and ACE for CLMBR-LR and end-to-end transformer (ETE) relative to ID

| **Task** | **Metric** | **Model** | **2013-2016** | **2017-2021** |
| --- | --- | --- | --- | --- |
| Hospital Mortality | Δ-AUROC | ETE | -0.003 | 0.004 |
|  |  | CLMBR-LR | 0.011 | 0.008 |
|  | Δ- AUPRC*_C_* | ETE | 0.061 | 0.014 |
|  |  | CLMBR-LR | 0.078 | 0.036 |
|  | Δ-ACE | ETE | -0.001 | **0.002** |
|  |  | CLMBR-LR | 0.001 | 0.013 |
| Long LOS | Δ-AUROC | ETE | 0.003 | -0.024 |
|  |  | CLMBR-LR | 0.002 | -0.026 |
|  | Δ- AUPRC*_C_* | ETE | 0.024 | **-0.014** |
|  |  | CLMBR-LR | 0.021 | -0.043 |
|  | Δ-ACE | ETE | 0.005 | **0.015** |
|  |  | CLMBR-LR | 0.006 | 0.038 |
| 30-day readmission | Δ-AUROC | ETE | -0.015 | -0.049 |
|  |  | CLMBR-LR | -0.016 | -0.058 |
|  | Δ- AUPRC*_C_* | ETE | 0.002 | -0.038 |
|  |  | CLMBR-LR | -0.011 | -0.057 |
|  | Δ-ACE | ETE | 0.001 | 0.012 |
|  |  | CLMBR-LR | -0.002 | 0.003 |
| ICU admission | Δ-AUROC | ETE | -0.009 | -0.037 |
|  |  | CLMBR-LR | -0.01 | -0.031 |
|  | Δ- AUPRC*_C_* | ETE | -0.048 | **-0.054** |
|  |  | CLMBR-LR | -0.104 | -0.184 |
|  | Δ-ACE | ETE | **-0.003** | **0.001** |
|  |  | CLMBR-LR | 0.007 | 0.008 |

Deltas were obtained by subtracting the model’s OOD performance by its in-distribution performance. Bolded values indicate the model between CLMBR-LR and ETE with significantly less performance degradation. Significance is determined based on 95% confidence intervals calculated from the distribution of differences obtained over 1000 iterations.

Abbreviations: AUROC: area under the receiver operating characteristics curve; AUPRC*_C_*: calibrated area under the precision recall curve; ACE: absolute calibration error; CLMBR: clinical language model-based representation; LR: logistic regression; LOS: length of stay; ICU: intensive care unit; ID: in-distribution; OOD: out-of-distribution.

**Supplementary Figure S2.** Correlation between the CLMBR pretraining performance and the performance of the downstream logistic regression models in each clinical prediction tasks. Performance for both the sequence model and the logistic regression model were measured using binary cross entropy loss. Each point in the scatter plot represents CLMBR’s performance in the validation set and its downstream logistic regression model’s performance in the test set. Each CLMBR model was pretrained using a different hyperparameter setting from the hyperparameter grid. Shaded error envelope represents the 95% confidence interval around the regression line.

Abbreviations: CLMBR: clinical language model-based representations; LOS: length of stay; ICU: intensive care unit.

**
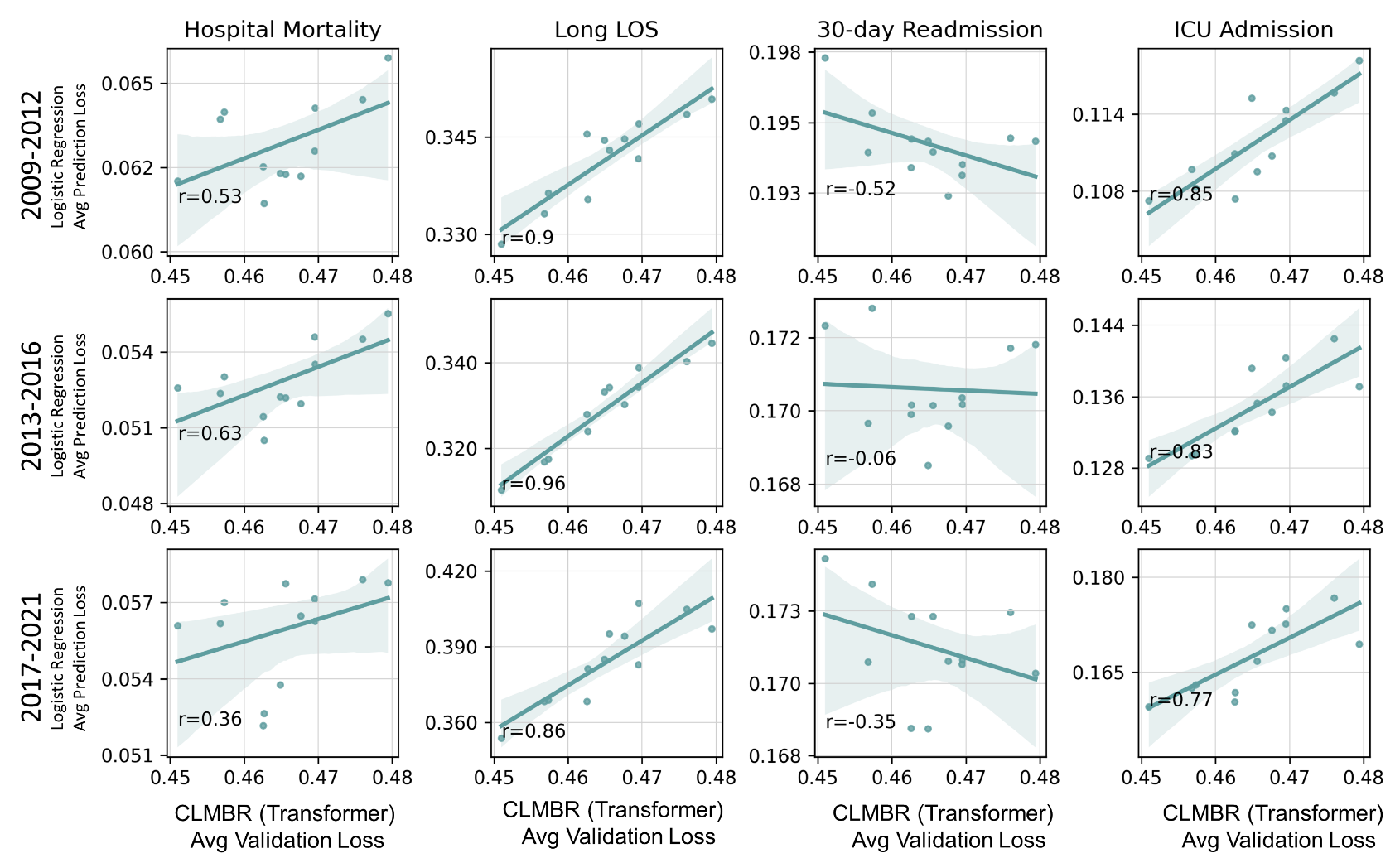
**

**Supplementary Table S6. ID and OOD Performance of GRU- and transformer-based CLMBR along increasing pretraining set size**

|  | | | Evaluation Year Group | | | | | | | |
| --- | --- | --- | --- | --- | --- | --- | --- | --- | --- | --- |
|  |  |  | 2009-2012 (ID) | | | | 2020-2021 (OOD) | | | |
|  |  |  | No. of Patients [Training Year Group] | | | | | | | |
| Task | Metric | CLMBR Architecture | 36K  [09-12*] | 610K  [09-12] | 1280K  [09-16] | 1820K  [09-19] | 36K  [09-12*] | 610K  [09-12] | 1280K  [09-16] | 1820K  [09-19] |
| Hospital Mortality | AUROC | GRU | **0.961** | 0.964 | 0.963 | 0.966 | **0.976** | 0.975 | 0.973 | 0.972 |
|  |  | Transformer | 0.946 | 0.958 | **0.975** | **0.975** | 0.956 | 0.971 | 0.978 | **0.979** |
|  | AUPRC*_C_* | GRU | **0.484** | **0.518** | 0.49 | 0.517 | **0.435** | 0.462 | 0.431 | 0.442 |
|  |  | Transformer | 0.369 | 0.39 | **0.59** | **0.603** | 0.338 | 0.418 | 0.501 | **0.527** |
|  | ACE | GRU | 0.003 | **0.002** | 0.002 | 0.002 | 0.006 | **0.005** | 0.005 | 0.003 |
|  |  | Transformer | 0.003 | 0.004 | 0.002 | 0.002 | 0.007 | 0.007 | 0.005 | 0.004 |
| Long LOS | AUROC | GRU | **0.88** | 0.878 | 0.888 | 0.887 | **0.87** | 0.867 | 0.889 | 0.898 |
|  |  | Transformer | 0.86 | **0.888** | **0.909** | **0.911** | 0.853 | 0.885 | 0.911 | 0.919 |
|  | AUPRC*_C_* | GRU | **0.668** | 0.667 | 0.687 | 0.688 | **0.644** | 0.646 | 0.681 | 0.705 |
|  |  | Transformer | 0.612 | **0.682** | **0.742** | **0.745** | 0.585 | **0.674** | **0.749** | **0.77** |
|  | ACE | GRU | **0.012** | 0.012 | 0.01 | 0.01 | 0.006 | 0.009 | 0.007 | 0.016 |
|  |  | Transformer | 0.019 | 0.015 | 0.006 | 0.004 | 0.008 | 0.013 | 0.01 | 0.012 |
| 30-day Readmission | AUROC | GRU | 0.801 | 0.798 | 0.803 | 0.801 | 0.789 | 0.783 | 0.793 | 0.791 |
|  |  | Transformer | 0.796 | 0.799 | 0.81 | **0.818** | 0.782 | 0.779 | 0.801 | **0.809** |
|  | AUPRC*_C_* | GRU | 0.235 | 0.245 | 0.238 | 0.245 | 0.223 | 0.21 | 0.205 | 0.217 |
|  |  | Transformer | 0.241 | 0.238 | **0.266** | 0.255 | 0.201 | 0.221 | **0.263** | **0.281** |
|  | ACE | GRU | 0.005 | 0.005 | 0.005 | 0.005 | 0.008 | 0.009 | 0.013 | 0.013 |
|  |  | Transformer | 0.007 | 0.007 | 0.004 | 0.004 | 0.009 | 0.01 | **0.007** | **0.007** |
| ICU Admission | AUROC | GRU | **0.947** | 0.947 | 0.953 | 0.954 | 0.917 | 0.915 | 0.924 | 0.928 |
|  |  | Transformer | 0.932 | 0.949 | **0.966** | **0.965** | 0.91 | **0.927** | **0.949** | **0.951** |
|  | AUPRC*_C_* | GRU | **0.63** | 0.635 | 0.652 | 0.652 | **0.597** | 0.585 | 0.617 | 0.648 |
|  |  | Transformer | 0.532 | 0.643 | **0.734** | **0.733** | 0.547 | **0.637** | **0.716** | **0.733** |
|  | ACE | GRU | 0.003 | 0.003 | 0.002 | 0.002 | **0.015** | 0.019 | 0.011 | **0.01** |
|  |  | Transformer | 0.002 | 0.004 | 0.002 | 0.002 | 0.019 | **0.01** | 0.011 | 0.013 |

Bolded values indicate the significantly better performing model between GRU- and transformer-based CLMBR. Significance is determined based on 95% confidence intervals calculated from the distribution of differences obtained over 1000 iterations.

Abbreviations: AUROC: area under the receiver operating characteristics curve; AUPRC*_C_*: calibrated area under the precision recall curve; ACE: absolute calibration error; CLMBR: clinical language model-based representation; LR: logistic regression; LOS: length of stay; ICU: intensive care unit; ID: in-distribution; OOD: out-of-distribution.

**Supplementary Table S7.** Scaling of GRU- and transformer-based CLMBR to pretraining set size

|  | | | Evaluation Year Groups | |
| --- | --- | --- | --- | --- |
| Task | Metric | CLMBR Architecture | 2009-2012 (ID) | 2020-2021(OOD) |
| Hospital Mortality | AUROC | GRU | 0.002 | -0.002 |
|  |  | Transformer | **0.017** | **0.013** |
|  | AUPRC*_C_* | GRU | 0.01 | 0 |
|  |  | Transformer | **0.152** | **0.11** |
|  | ACE | GRU | -0.001 | -0.002 |
|  |  | Transformer | -0.002 | -0.002 |
| Long LOS | AUROC | GRU | 0.005 | 0.018 |
|  |  | Transformer | **0.029** | **0.037** |
|  | AUPRC*_C_* | GRU | 0.013 | 0.034 |
|  |  | Transformer | **0.073** | **0.1** |
|  | ACE | GRU | -0.001 | 0.005 |
|  |  | Transformer | **-0.01** | 0.001 |
| 30-day Readmission | AUROC | GRU | 0 | 0.002 |
|  |  | Transformer | **0.012** | **0.017** |
|  | AUPRC*_C_* | GRU | 0.003 | -0.005 |
|  |  | Transformer | 0.013 | **0.049** |
|  | ACE | GRU | 0 | 0.003 |
|  |  | Transformer | **-0.003** | **-0.002** |
| ICU Admission | AUROC | GRU | 0.005 | 0.007 |
|  |  | Transformer | **0.019** | **0.025** |
|  | AUPRC*_C_* | GRU | 0.012 | 0.031 |
|  |  | Transformer | **0.121** | **0.112** |
|  | ACE | GRU | -0.001 | -0.004 |
|  |  | Transformer | 0 | -0.003 |

Values indicate the slope of the regression line fitted on the performance along pretraining set size and can be interpreted as the estimated change in performance (AUROC, AUPRC*_C_*, ACE) per increase in 1 million patients in the pretraining set. Bolded values indicate the more scalable architecture between GRU and transformer. Significance is determined based on 95% confidence intervals calculated from the distribution of differences obtained over 1000 iterations.

Abbreviations: AUROC: area under the receiver operating characteristics curve; AUPRC*_C_*: calibrated area under the precision recall curve; ACE: absolute calibration error; CLMBR: clinical language model-based representation; LR: logistic regression; LOS: length of stay; ICU: intensive care unit; ID: in-distribution; OOD: out-of-distribution.

**Supplementary GRU Experiment.** Ablation of CLMBR architecture – replacement of Transformer with GRU.

1. Methods
2. GRU hyperparameter grid
3. Selected GRU hyperparameter values
4. Selected logistic regression hyperparameter values
5. CLMBR_GRU_-LR vs. Count-LR
6. CLMBR_GRU_-LR vs. ETE_GRU_
7. Correlation between CLMBR pretraining performance and the performance of downstream logistic regression models
8. Discussion

**Supplementary GRU Experiment I. Methods**

In this ablation experiment, we replaced transformer (CLMBR_TRANS_) with gradient recurrent unit (CLMBR_GRU_) as the CLMBR architecture. For fair comparison, we also used GRU as the architecture for the end-to-end models (ETE_GRU_). Besides the architecture, all procedures remained the same with the exception that positional encoding based on patient day and age are not needed for CLMBR_GRU_ and so are excluded.

Training of CLMBR_GRU_ and ETE_GRU_ followed the same procedure as CLMBR_TRANS_ and ETE_TRANS._ The tables in Supplementary Experiment 1 II and III list the hyperparameters in the grid search and the selected hyperparameter values, respectively.

We used logistic regression as the classification head for each downstream clinical prediction task. Training and evaluation followed the same procedure as CLMBR_TRANS_-LR in the main experiment. Table in Supplementary Experiment 1 IV lists the selected logistic regression hyperparameter values.

**Supplementary GRU Experiment II. GRU hyperparameters**

| **Architecture** | **Hyperparameter** | **Task** | **Values** |
| --- | --- | --- | --- |
| GRU ^a^ | Learning rate | Sequence modeling (CLMBR pretraining) & end-to-end | 0.01, 0.001, 0.0001 |
| GRU | L2 | Sequence modeling (CLMBR pretraining) & end-to-end | 0.1, 0.01, 0.001 |
| GRU | Batch size | Sequence modeling (CLMBR pretraining) | 2000, 4000 |

CLMBR models were trained up to 10,000 epochs and stopped early if no improvement in the validation set was observed for 100 epochs. End-to-end models were trained for 50 epochs.

^a^ All GRU architectures consisted of one layer. Code dropout was set at 0.2.

Abbreviations: CLMBR: clinical language model-based representations; GRU: gated recurrent unit.

**Supplementary GRU Experiment III**. **Selected model hyperparameters for CLMBR and end-to-end models**

| **Architecture** | **Task** | **Hyperparameter** | **Value** |
| --- | --- | --- | --- |
| GRU | Sequence modeling (CLMBR pretraining) | Learning rate | 0.001 |
| GRU | Sequence modeling  (CLMBR pretraining) | Batch size | 2000 |
| GRU | Sequence modeling  (CLMBR pretraining) | L2 | 0.01 |
| GRU | Hospital Mortality | Learning rate | 0.001 |
| GRU | Hospital Mortality | L2 | 0.1 |
| GRU | Long LOS | Learning rate | 0.001 |
| GRU | Long LOS | L2 | 0.1 |
| GRU | ICU Admission | Learning rate | 0.001 |
| GRU | ICU Admission | L2 | 0.01 |
| GRU | 30d readmission | Learning rate | 0.0001 |
| GRU | 30d readmission | L2 | 0.1 |

Abbreviations: CLMBR: clinical language model-based representations; GRU: gated recurrent unit; ICU: intensive care unit; LOS: length of stay

**Supplementary GRU Experiment IV**. **Selected model hyperparameter for logistic regression**

| **Featurization** | **Task** | **L2 regularization strength** |
| --- | --- | --- |
| CLMBR | Hospital Mortality | 0.1 |
| CLMBR | Long LOS | 0.01 |
| CLMBR | ICU Admission | 0.1 |
| CLMBR | 30d readmission | 0.01 |

^a^Smaller values indicate stronger regularization. Search was conducted over values ranging from 10^-6^ to 10^2^ in powers of 10

Abbreviations: CLMBR: clinical language model-based representations; ICU: intensive care unit; LOS: length of stay

**Supplementary GRU Experiment V**. Performance of GRU-based CLMBR-LR (CLMBR_GRU_-LR) vs. Count-LR in 2009-2012 (09-12), 2013-2016 (13-16), and 2017-2021 (17-21). Like CLMBR_TRANS_-LR, CLMBR_GRU_-LR displayed better discrimination performance in-distribution (ID) and out-of-distribution (OOD) in 3 out of 4 clinical prediction tasks. The shaded region for each model indicates performance degradation in OOD year groups relative to the ID year group. A larger shaded region indicates more degradation of performance. Error bars indicate 95% confidence interval obtained from 1000 bootstrap iterations.

Abbreviations: AUROC: area under the receiver operating characteristics curve; AUPRC*_C_*: calibrated area under the precision recall curve; ACE: absolute calibration error; LOS: length of stay; ICU: intensive care unit; CLMBR: clinical language model-based representation; LR: logistic regression; GRU: gradient-recurrent unit.


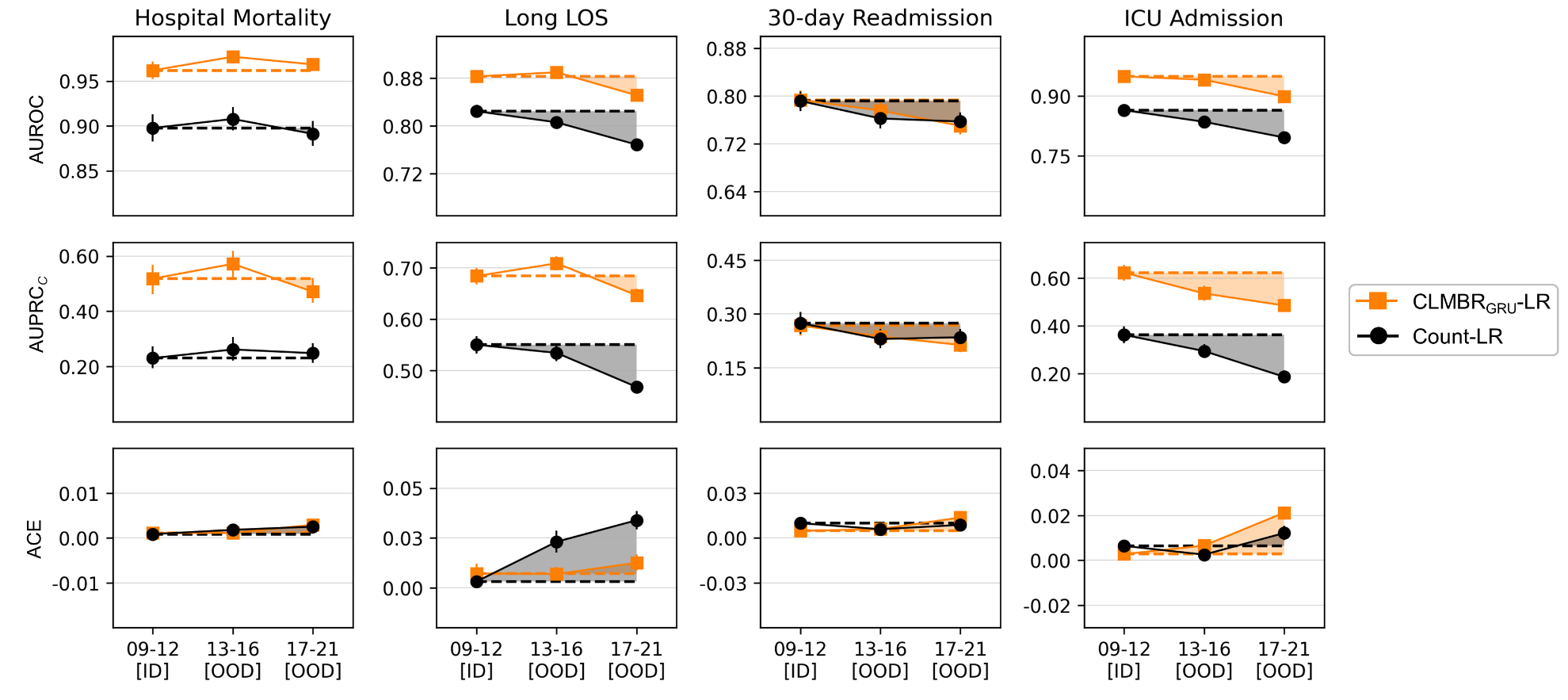


**Supplementary GRU Experiment VI**. Performance of GRU-based CLMBR-LR (CLMBR_GRU_-LR) vs. GRU-based ETE (ETE_GRU_) in 2009-2012 (09-12), 2013-2016 (13-16), and 2017-2021 (17-21). CLMBR_GRU_-LR performed as well as or better than ETE. The shaded region for each model indicates performance degradation in OOD year groups relative to the ID year group. A larger shaded region indicates more degradation of performance. Error bars indicate 95% confidence interval obtained from 1000 bootstrap iterations.

Abbreviations: AUROC: area under the receiver operating characteristics curve; AUPRC*_C_*: calibrated area under the precision recall curve; ACE: absolute calibration error; LOS: length of stay; ICU: intensive care unit; CLMBR: clinical language model-based representation; LR: logistic regression; GRU: gradient-recurrent unit.


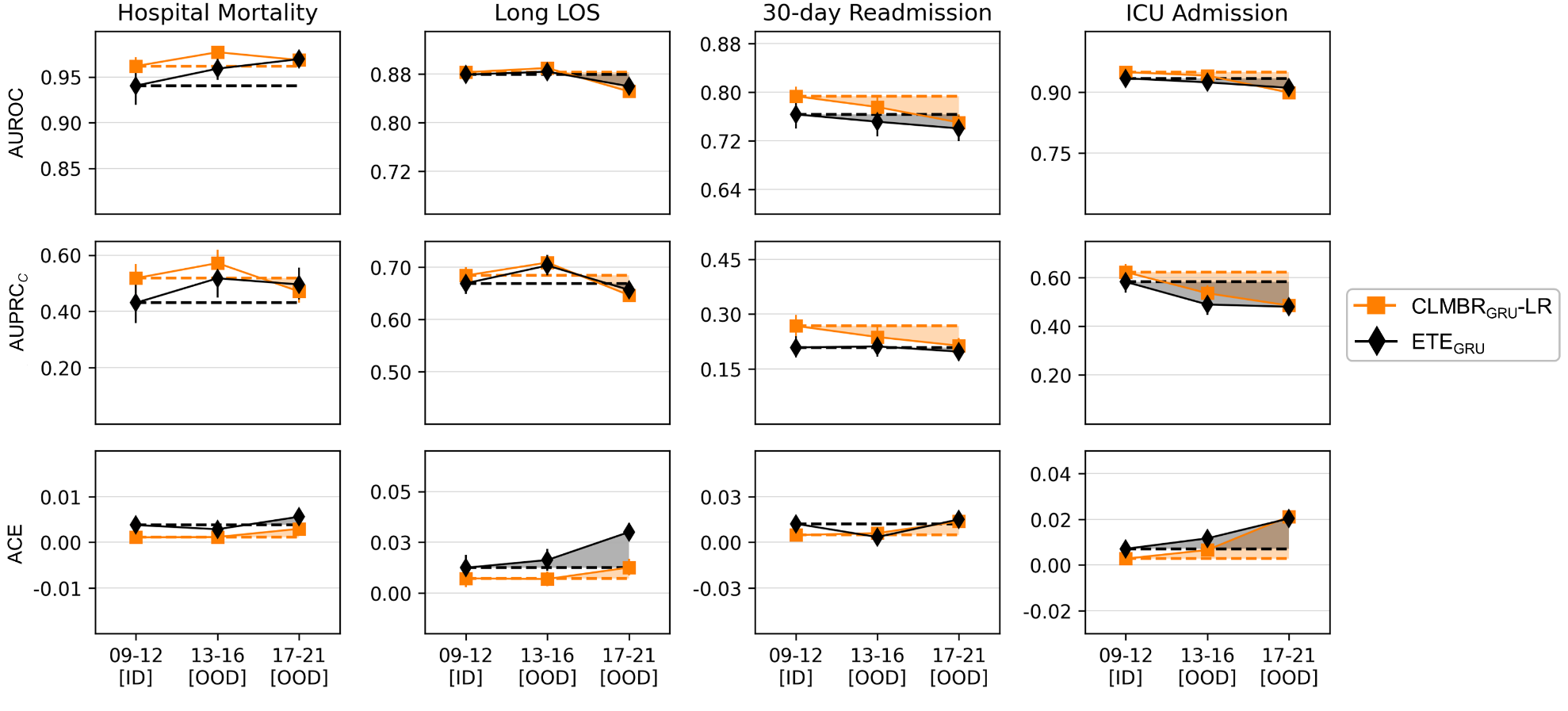


**Supplementary GRU Experiment VII**. Correlation between the CLMBR (GRU) pretraining performance and the performance of the downstream logistic regression models in each clinical prediction tasks. Performance for both the sequence model and the logistic regression model were measured using binary cross entropy loss. Each point in the scatter plot represents CLMBR’s performance in the validation set and its downstream logistic regression model’s performance in the test set. Each CLMBR model was pretrained using a different hyperparameter setting from the hyperparameter grid. Shaded error envelope represents the 95% confidence interval around the regression line.

Abbreviations: CLMBR: clinical language model-based representations; LOS: length of stay; ICU: intensive care unit.


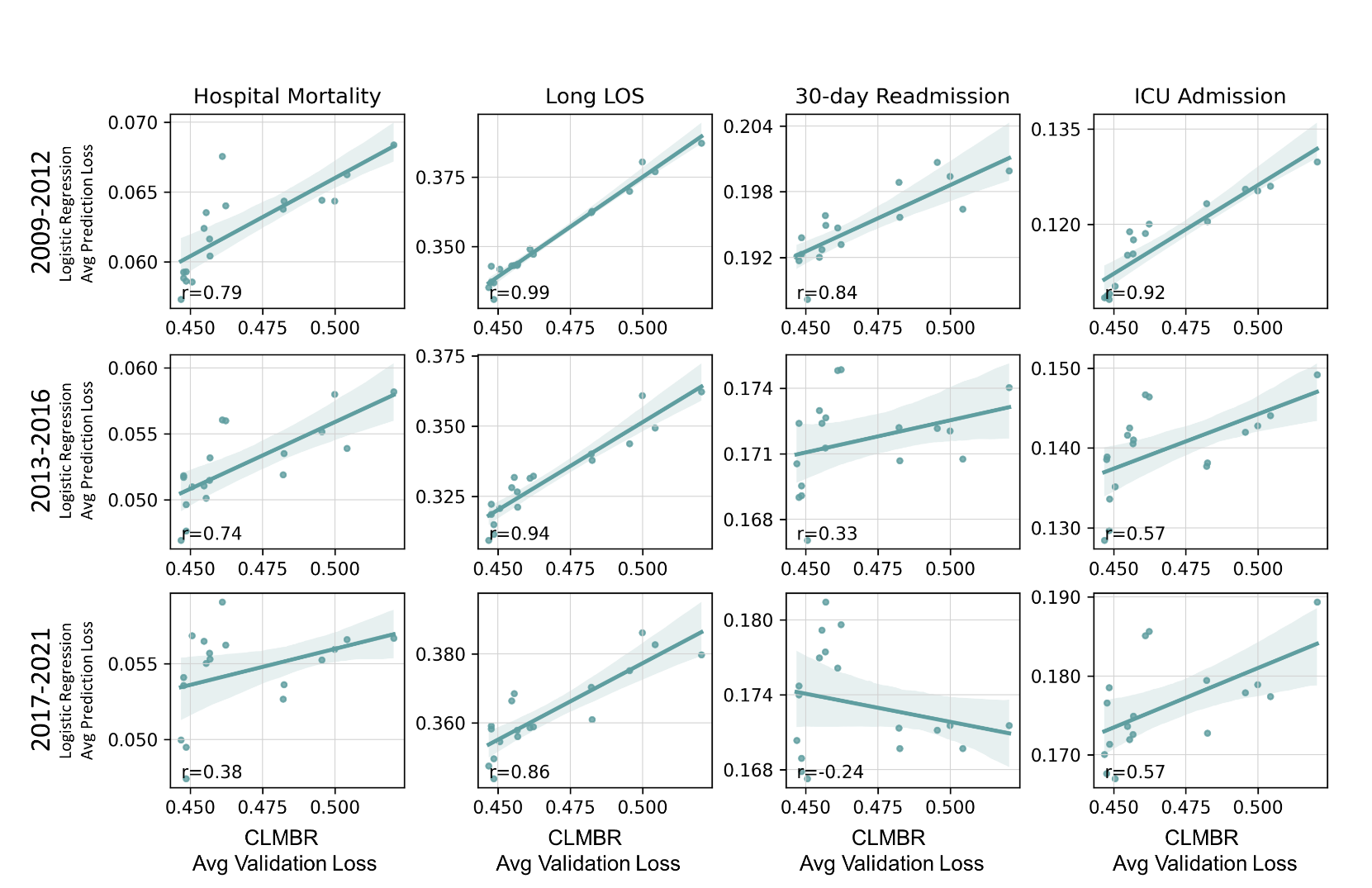


**Supplementary GRU Experiment VIII. Discussion**

We found that GRU-based CLMBR (CLMBR_GRU_) performed at similar levels as transformer-based CLMBR (CLMBR_TRANS_). Their downstream models (CLMBR_GRU_-LR) also performed qualitatively similarly in ID and OOD years. One notable difference is that the gap between CLMBR_TRANS_-LR and ETE_TRANS_ seems larger (Figure 4) than CLMBR_GRU_-LR and ETE_GRU_ (Supplementary Experiment 1 V). We hypothesize that this is due to GRU being better with smaller training sets than the transformer. Despite this, ETE_TRANS_ still seemed to have better discrimination performance than count-LR. Correlations between CLMBR_GRU_ validation loss and LR validation loss follow a similar pattern as CLMBR_TRANS_.

Overall, with a pretraining set size of ~600,000 (80% training, 20% validation), CLMBR_GRU_ reaches parity with CLMBR_TRANS_.

**Supplementary LightGBM Experiment.** Ablation of classification head – replacement of logistic regression with LightGBM

1. Methods
2. Selected LightGBM hyperparameter values
3. The impact of temporal dataset shift on the performance of LightGBM models trained on count-based representations
4. CLMBR-LightGBM vs. Count-LightGBM
5. Discussion

**Supplementary LightGBM Experiment I. Methods.**

In this ablation experiment we replaced logistic regression with light gradient boosting machines (LightGBM) as the classification head for each clinical prediction model. The construction of patient representations followed the same procedure as count-LR for count-based features, and CLMBR-LR for CLMBR. We used transformer-based CLMBR from the main experiment to construct CLMBR-based patient representations.

Training and evaluation of count-LightGBM and CLMBR-LightGBM followed the same procedure as count-LR and CLMBR-LR. Grid search for each LightGBM was conducted over learning rate (0.1, 0.2, 0.01), number of leaves (100, 300), and boosting type (gbdt, dart, goss). The number of trees for gbdt and goss was determined based on performance in the task-specific validation set via early stopping. The number of trees for dart was fixed at 1000.

**Supplementary LightGBM Experiment II**. Selected light gradient boosting machine (LightGBM) hyperparameter values

| **Featurization** | **Task** | **Hyperparameter^a^ Values** |
| --- | --- | --- |
| Count-based | Hospital Mortality | **lr**: 0.01; **num_leaves**: 100; **boosting_type**: goss; **n_estimators**: 312 |
| Count-based | Long LOS | **lr**: 0.01; **num_leaves**: 100; **boosting_type**: gbdt; **n_estimators**: 650 |
| Count-based | ICU Admission | **lr**: 0.01; **num_leaves**: 100; **boosting_type**: goss; **n_estimators**: 427 |
| Count-based | 30d readmission | **lr**: 0.01; **num_leaves**: 100; **boosting_type**: goss; **n_estimators**: 366 |
| CLMBR (Transformer) | Hospital Mortality | **lr**: 0.01; **num_leaves**: 100; **boosting_type**: goss; **n_estimators**: 249 |
| CLMBR (Transformer) | Long LOS | **lr**: 0.01; **num_leaves**: 100; **boosting_type**: goss; **n_estimators**: 530 |
| CLMBR (Transformer) | ICU Admission | **lr**: 0.01; **num_leaves**: 100; **boosting_type**: goss; **n_estimators**: 326 |
| CLMBR (Transformer) | 30d readmission | **lr**: 0.01; **num_leaves**: 100; **boosting_type**: goss; **n_estimators**: 186 |

**Supplementary LightGBM Experiment III**. The impact of temporal dataset shift on the performance (AUROC, AUPRC*_C_*, and Calibration measured using ACE) of Light Gradient Boosting Machine-based clinical prediction models (LightGBM) trained on count-based patient representations (count-LightGBM). Shaded regions indicate time windows in which performance in out-of-distribution (OOD) years (2013-2021) is worse (red) or better (green) than performance in the in-distribution year group (2009-2012). A Larger red shaded region indicates more degradation relative to the model’s in-distribution performance. Oracle models were trained and evaluated on each of the OOD years. Error bars indicate 95% confidence interval obtained from 1000 bootstrap iterations. Like count-LR, the impact of temporal distribution shift on count-LightGBM is larger for Long LOS and Admission to ICU prediction tasks.

Abbreviations: AUROC: area under the receiver operating characteristics curve; AUPRC*_C_*: calibrated area under the precision recall curve; ACE: absolute calibration error; LOS: length of stay; ICU: intensive care unit.


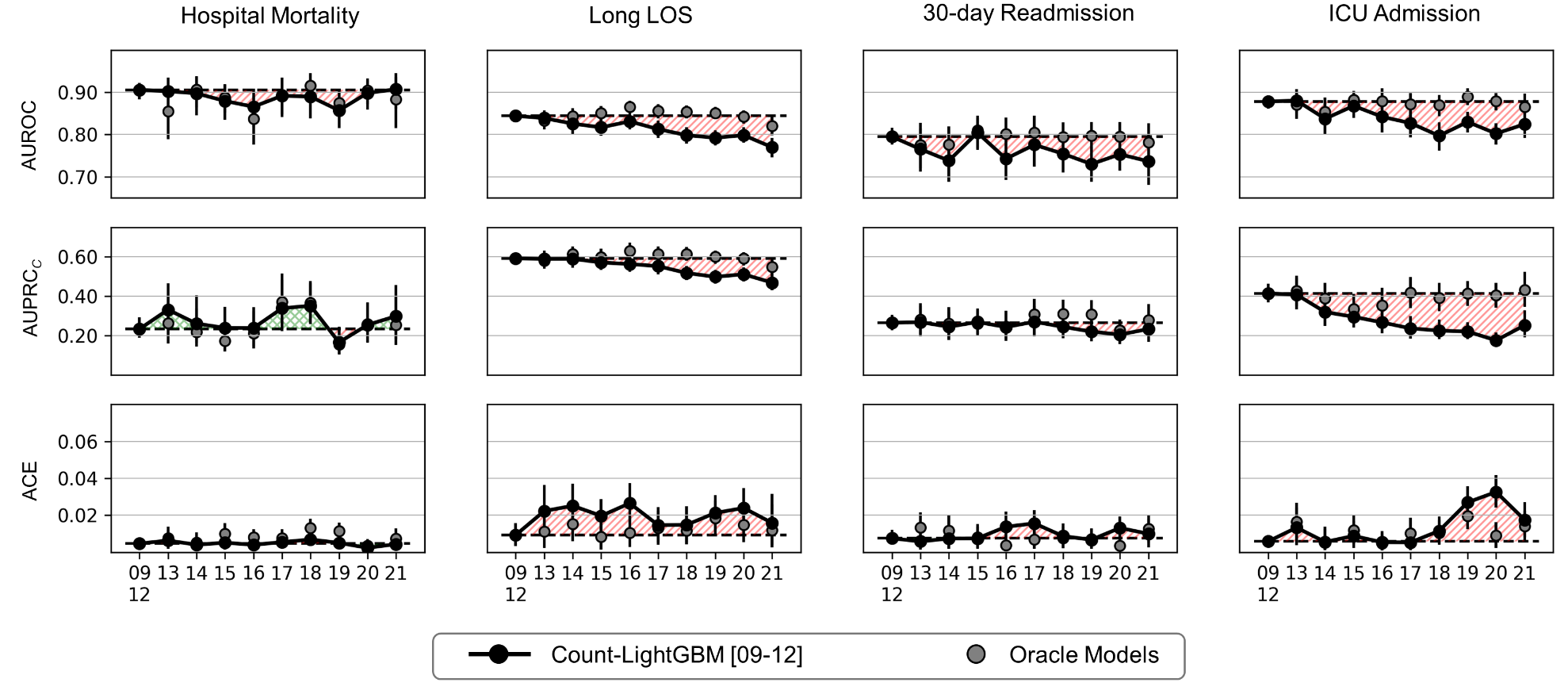


**Supplementary LightGBM Experiment IV**. Performance of CLMBR-LightGBM vs. Count- LightGBM in 2009-2012 (09-12), 2013-2016 (13-16), and 2017-2021 (17-21). Like the comparison between CLMBR-LR and count-LR, CLMBR-LightGBM had better discrimination performance in-distribution (ID) and out-of-distribution (OOD) than count-LightGBM in 3 out of 4 clinical prediction tasks. The shaded region for each model indicates performance degradation in OOD year groups relative to the ID year group. A larger shaded region indicates more degradation of performance. Error bars indicate 95% confidence interval obtained from 1000 bootstrap iterations.

Abbreviations: AUROC: area under the receiver operating characteristics curve; AUPRC*_C_*: calibrated area under the precision recall curve; ACE: absolute calibration error; LOS: length of stay; ICU: intensive care unit; CLMBR: clinical language model-based representation; LightGBM: light gradient boosting machines.


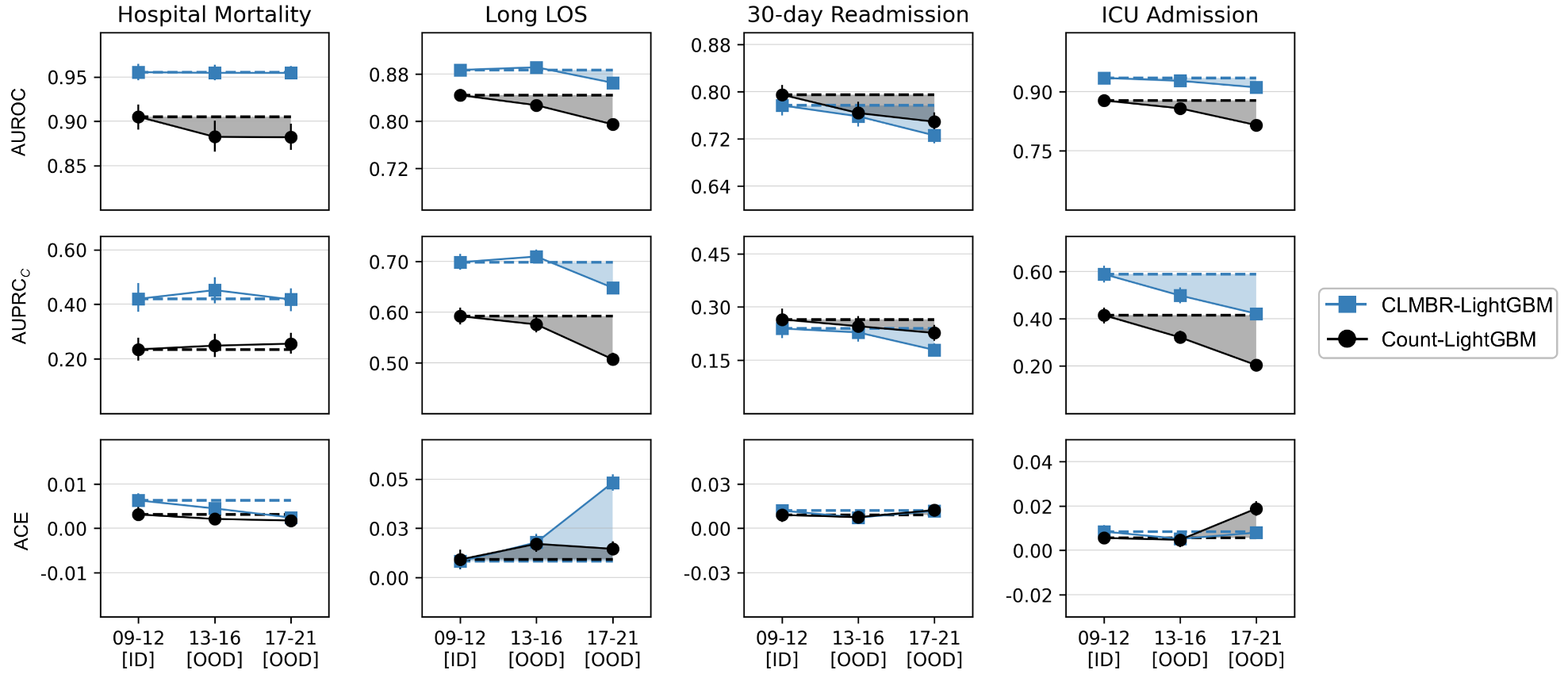


**Supplementary LightGBM Experiment V.** **Discussion.**

We saw similar variations in performance in count-LightGBM as count-LR with the largest deterioration of performance observed in long LOS and ICU admission prediction tasks (Supplementary Experiment 2 III). Supplementary Experiment 2 IV also displayed similar patterns as Figure 4. That is, in general, CLMBR-LightGBM displayed better discrimination performance than count-LightGBM (except in 30-day readmission prediction) in both ID and OOD test sets.

Based on these results, we recommend using logistic regression over LightGBM as classification heads for CLMBR due to its simplicity and efficiency.
